# Recent advances in CMOS-compatible synthesis and integration of 2D materials

**DOI:** 10.1186/s40580-025-00478-1

**Published:** 2025-02-15

**Authors:** Ajit Kumar Katiyar, Jonggyu Choi, Jong-Hyun Ahn

**Affiliations:** School of Electrical and Electronic Engineering, Yonsei University, Seoul, 03722 Republic of Korea

**Keywords:** 2D materials, CMOS devices, Flexible electronics, M3D integration, TMDs

## Abstract

The upcoming generation of functional electronics in the era of artificial intelligence, and IoT requires extensive data storage and processing, necessitating further device miniaturization. Conventional Si CMOS technology is struggling to enhance integration density beyond a certain limit to uphold Moore’s law, primarily due to performance degradation at smaller dimensions caused by various physical effects, including surface scattering, quantum tunneling, and other short-channel effects. The two-dimensional materials have emerged as highly promising alternatives, which exhibit excellent electrical and mechanical properties at atomically thin thicknesses and show exceptional potential for future CMOS technology. This review article presents the chronological progress made in the development of two-dimensional materials-based CMOS devices with comprehensively discussing the advancements made in material production, device development, associated challenges, and the strategies to address these issues. The future prospects for the use of two-dimensional materials in functional CMOS circuitry are outlooked, highlighting key opportunities and challenges toward industrial adaptation.

**Graphical Abstract:**

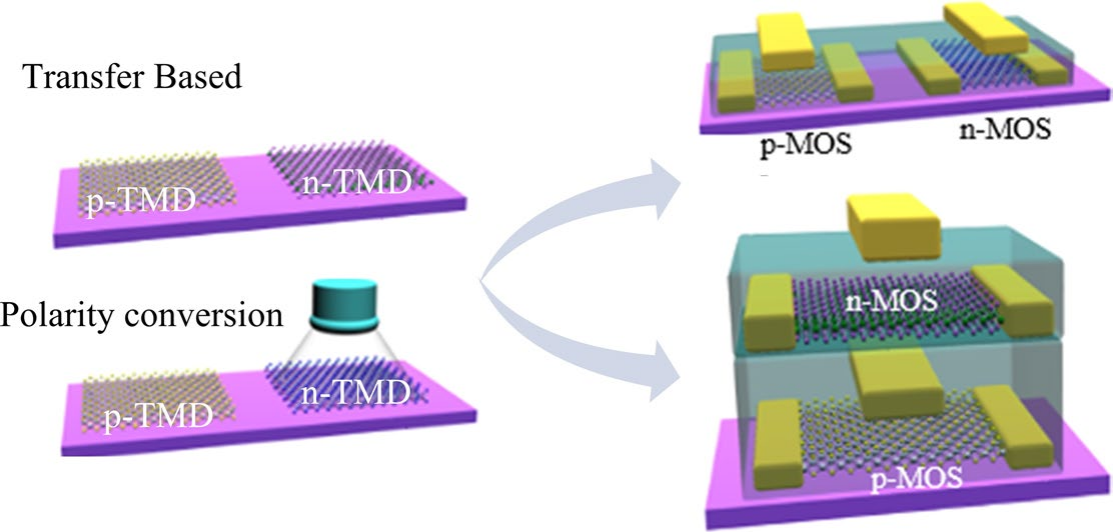

## Introduction

The complementary metal–oxide–semiconductor (CMOS) devices have served as the backbone of the microelectronic industry for the past few decades. The success of conventional CMOS technology lies in its complementary design, in which both n-type and p-type transistors are created on the same Si-semiconductor block which ensures effective functionality with optimal power efficiency. As the demand for higher density in electronic units grows within ever-evolving high-performance microprocessors, efforts to scale devices have primarily focused on reducing channel length and employing three-dimensional fin field-effect transistor (FinFET) and gate all around (GAA) FET structures. However, when the channel length decreases below few nm (the size of the depletion layer at source and drain junctions), the short channel effect arises which hinders the effective operation. Moreover, using Si fins and nanosheets with a thickness below ~ 3 nm leads to a drastic degradation in the charge carrier mobility due to increased scattering at the channel/dielectric interface [1, 2]. Due to the appearance of these unavoidable physical phenomena under low-dimensions, the conventional CMOS technology faces substantial challenges in increasing the integration density with size reduction. Meeting the growing demand for more powerful computing systems in the era of internet-of-things (IoT) and artificial intelligence (AI) necessitates the exploration of alternative semiconductor materials with superior scaling capabilities.

**Fig. 1:**
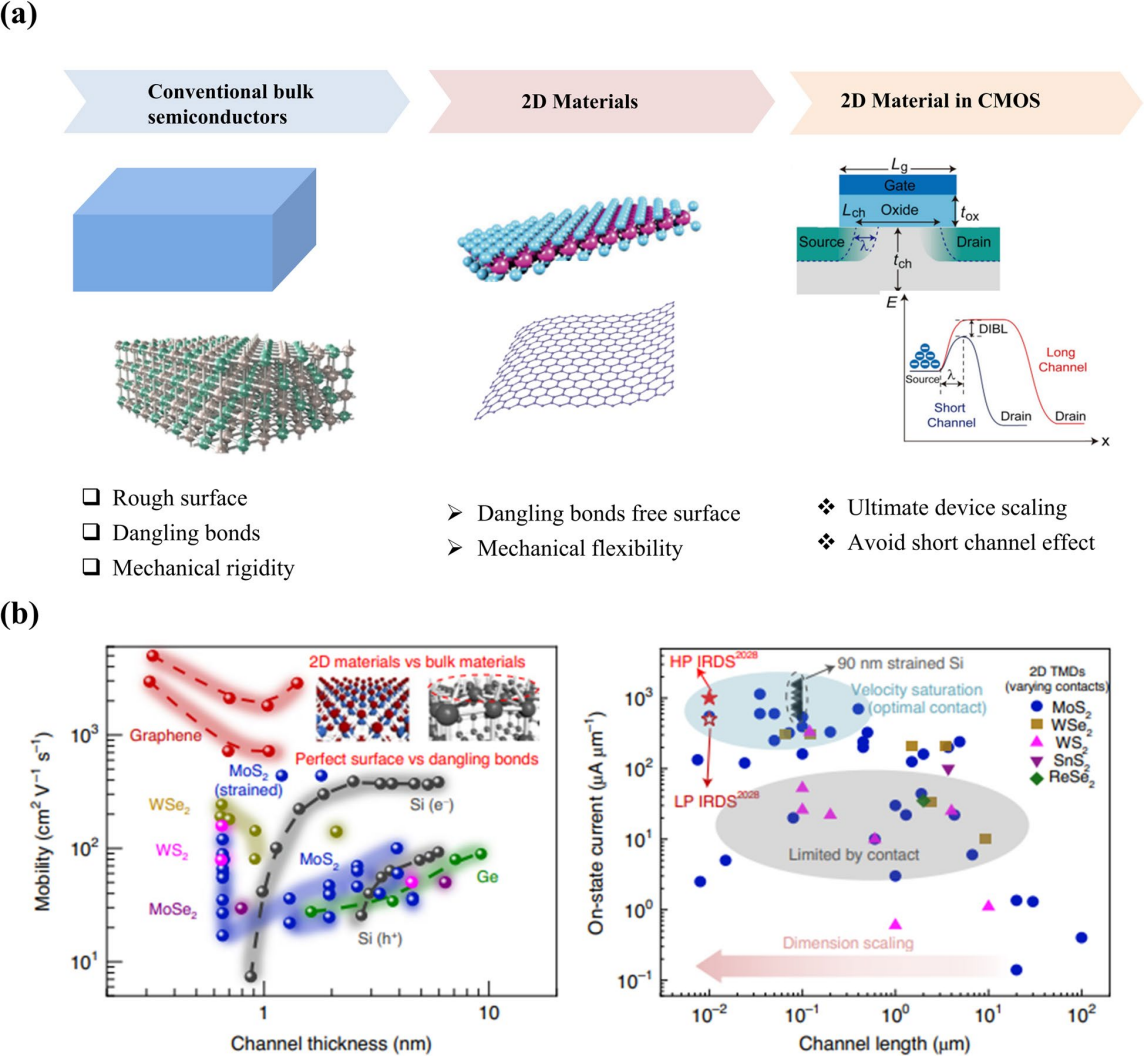
Merits of 2D materials over conventional bulk materials toward the development of ultra-scaled electronic devices. **a** Schematic representations of the bulk semiconductor and 2D materials. Corresponding merits and de-merits are highlighted below. Schematic representation of the typical MOS-FET structure. The band diagram of the long- and short-channel FETs showing the drain-induced barrier lowering (DIBL) occurring in bulk semiconductors-based MOS-FETs. Reproduced with permission [10]. Copyright 2022*,* Elsevier. **b** Carrier mobility of various representative channel materials including bulk Si, Ge, and various 2D materials (left panel). The right panel shows the on-current levels of FETs fabricated with various TMDs. Reproduced with permission [9]. Copyright 2022, Springer Nature

**Fig. 2:**
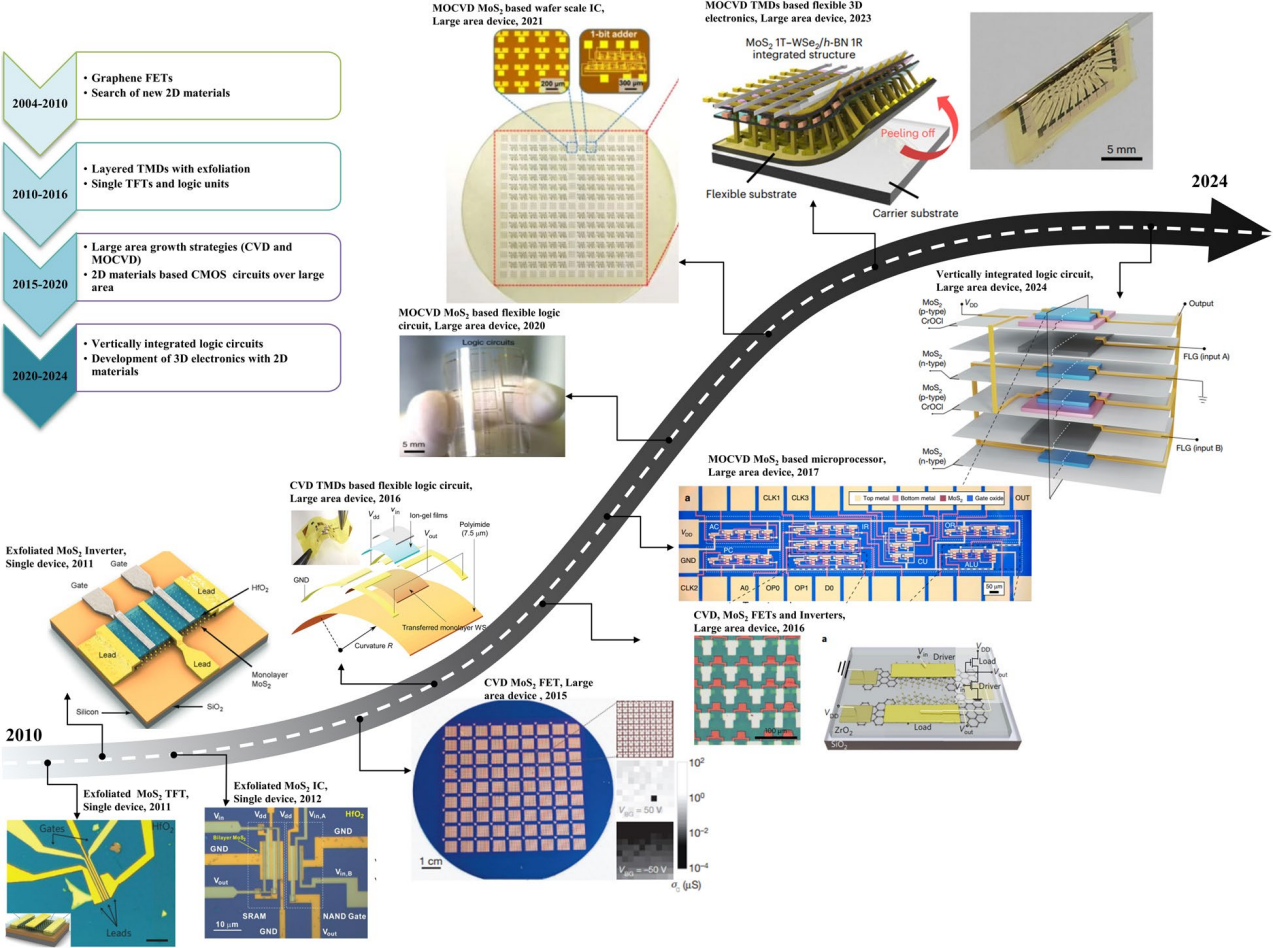
Roadmap showing the chronological development of 2D materials-based CMOS devices. From left to right; Microscopic image of a single thin film transistor fabricated with exfoliated MoS_2_ flake. Reproduced with permission [4]. Copyright 2011, Springer Nature. Optical image of an integrated circuit fabricated with mechanically exfoliated single-layer MoS_2_. The developed device consists of two nearby designed transistors controlled with HfO_2_ gate dielectric. Reproduced with permission [21]. Copyright 2011, American Chemical Society*.* Image of the NAND gate and the SRAM devices fabricated with exfoliated bilayer MoS_2_. Both of the devices were designed on the same MoS_2_ flake. Reproduced with permission [20]. Copyright 2012, American Chemical Society*.* FET arrays fabricated with CVD-grown large are MoS_2_. Reproduced with permission [19]. Copyright 2015, Springer Nature. CVD-grown TMDs-based flexible logic circuits. Reproduced with permission [18]. Copyright 2016, Wiley–VCH GmbH. CVD-grown MoS_2_-based FETs array and inverters. The two n-type transistors were integrated together to realize an inverter. Reproduced with permission [17]. Copyright 2016, Springer Nature. The microprocessor developed with MOCVD grown large are MoS_2_ films. Reproduced with permission [16]. Copyright 2017, Springer Nature*.* Integration of multistage MoS_2_ transistors to realize various logic devices such as inverters, NOR-, NAND-gates, AND-gates, ring oscillators, and SRAMs over large area flexible substrates. Reproduced with permission [15]. Copyright 2020, Springer Nature. Development of wafer scale integrated circuits over a 2-inch MoS_2_ wafer. Reproduced with permission [14]. Copyright 2021, Springer Nature. 2D materials based flexible M3D device. Reproduced with permission [13]. Copyright 2023, Springer Nature. M3D electronics CMOS electronics developed with combining 2D materials based n- and p-type FETs in vertical architecture. Reproduced with permission [12]. Copyright 2024, Springer Nature

After the experimental demonstration of the gate-controlled electrical conductivity in graphene flakes two decades ago [3], 2D materials have attracted significant research interest, prompting the scientific community to actively seek out new semiconducting 2D materials for use as ultrathin channel materials in electronic applications. Notably, many 2D materials, especially transition-metal dichalcogenides (TMDs), have emerged as outstanding ultrathin semiconductors with impressive electrical properties, delivering highly effective gate-controlled performance even at the extreme limits of device downscaling [4–8]. Their dangling bonds free surface and atomically thin thickness enable the fabrication of electronic devices with ultimate size miniaturization while avoiding the flaws associated with short channel and surface scattering effects which usually occur with downscaling conventional bulk semiconductors (Fig. 1a). With the decrease in the channel thickness of the conventional bulk semiconductors below 4 nm, the charge carrier mobility decreases drastically mainly due to the existence of the surface imperfections. Whereas, the dangling bonds-free surface of 2D materials maintains the level of mobility even at atomic scale thickness without any degradation (Fig. 1b) [9]. Consequently, 2D materials have been extensively explored as alternative semiconductors for channel materials in CMOS devices, aiming to advance device downscaling into both traditional scaling and new, innovative applications. In 2011, Radisavljevic et al. demonstrated the first transistor with exfoliated monolayer molybdenum disulfide (MoS_2_) flake which exhibited a semiconducting nature with a band gap of ~ 1.8 eV [4]. Right after this work, the same group reported the development of the integrated circuits (ICs) by fabricating two single-layer MoS_2_ transistors connected in series and demonstrated simple logic operations such as inverter and NOR logic [11]. As summarized in Fig. 2, with the rapid advancement in the chemical vapor deposition (CVD) and metal organic chemical vapor deposition (MOCVD) techniques for the large area production of 2D materials, several prototype examples, such as integrated circuits and microprocessors on both rigid and flexible platforms have been demonstrated [4, 12–21]. Additionally, recent advancements in three-dimensional (3D) heterogeneous integration technology have facilitated the creation of vertically integrated monolithic 3D (M3D) electronics based entirely on 2D materials, resulting in a higher density of electronic units and superior bandwidth performance compared to what is achievable with lateral integrations [12, 13, 22–27].

Despite the rapid advancements in 2D technology in terms of material production and their implementation toward the next generation electronics, the commercial deployment of 2D materials is still in the early stages. 2D materials encounter several inherent limitations and challenges that impede their widespread use, necessitating additional efforts to overcome these obstacles for future advancement. For instance, high contact resistance, the challenge of depositing high-quality dielectric materials on the surface of dangling bond-free 2D materials, and the complexity of selective doping for local p- or n-type conversion are significant obstacles in 2D materials-based CMOS electronics. Moreover, the low-temperature production of defect-free 2D materials is crucial for developing high-performance electronics, yet producing large-area 2D materials at a low thermal budget remains a challenge.

In this review article, we present the progress made toward developing 2D materials-based CMOS electronics while discussing the advancements made in material production and device fabrication technology in detail. The current challenges in exploiting 2D materials in CMOS technology and the strategies to mitigate these challenges are highlighted. In addition, the studies demonstrating the mechanical merits of 2D semiconductors in developing high-performance flexible CMOS units are reviewed. Furthermore, the recent advancement in 2D materials-based vertically integrated M3D devices demonstrating the breakthrough examples of high-density CMOS devices with high operational bandwidth are discussed in detail. Finally, we outlooked the future development roadmaps for capitalization of 2D materials in functional CMOS circuitry highlighting the key opportunities and challenges toward the industrial adaptation of 2D technology.

## Synthesis of 2D materials for CMOS devices

**Fig. 3:**
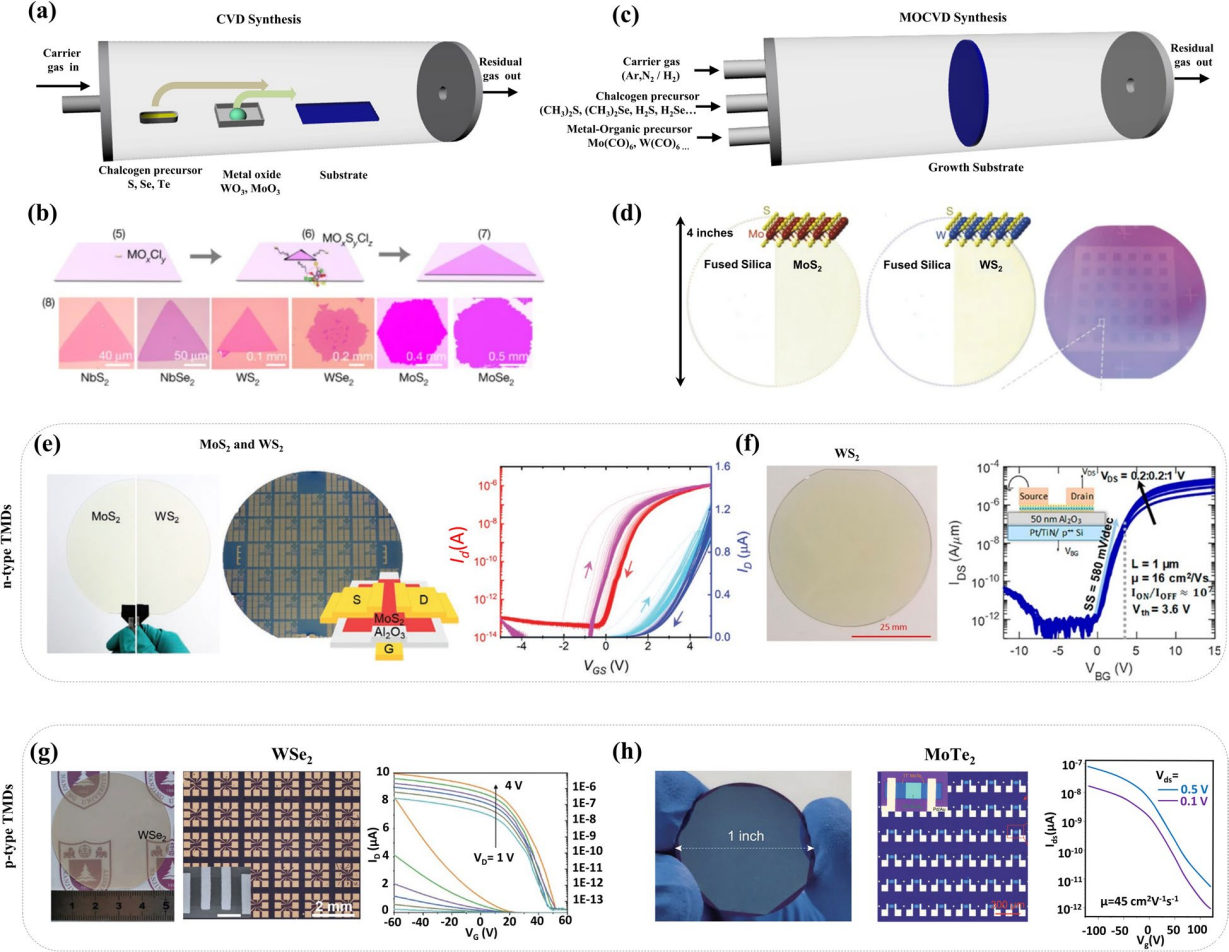
Synthesis of p- and n-type 2D semiconductors for CMOS electronics. **a** Schematic representation of a typical CVD system. **b** CVD growth mechanism for synthesizing the 2D atomic layers and optical microscopic images of various monolayer TMD crystals synthesized using the CVD approach. Reproduced with permission [62]. Copyright 2018, Springer Nature. **c** Schematic diagram representing a typical MOCVD growth chamber generally utilized to grow the wafer scale TMDs. A precise flow of metal–organic and chalcogen percusses is inserted into the growth chamber using mass flow controllers. **d** Photographic images of MoS_2_ and WS_2_ monolayers synthesized on fused silica substrates. The right image shows the patterned MoS_2_ film after transferring it to a 4-inch SiO_2_/Si substrate. Reproduced with permission [19]. Copyright 2015, Springer Nature. **e** Photograph of n-type monolayer TMDs, MoS_2_ and WS_2_, grown on 6-inch quartz wafers and a batch of n-channel TFT arrays fabricated with grown MoS_2_ over a large area. The right panel shows the transfer curves of 900 FETs having channel width and channel length of 50 μm, and 10 μm, respectively. Reproduced with permission [75]. Copyright 2020, Wiley‐VCH GmbH. **f** Photograph of WS_2_ monolayer film grown on a 2-inch sapphire substrate (left panel). The right panel shows the transfer characteristics of a representative WS_2_ exhibiting a typical n-type nature. Reproduced with permission [76]. Copyright 2021, American Chemical Society*.* **g** Optical image of a p-type WSe_2_, grown over a 2-inch sapphire wafer (left). The optical microscopic image of fabricated FET arrays (middle), and corresponding electrical characteristics clearly exhibit the p-nature (right). Reproduced with permission [77]. Copyright 2023, Wiley‐VCH GmbH*.* **h** Photograph and microscopic image of 2H MoTe_2_ synthesized on 1-inch wafer and 1 T′/2H/1 T′ heterophase MoTe_2_ based FET array fabricated with a phase-engineering method. The right panel shows the obtained electrical characteristics of the fabricated devices [78]. Copyright 2021, American Association for the Advancement of Science

Novoselov and Geim opened the era of 2D materials-based electronics by experimentally demonstrating the electric field-driven tuning of electrical conductivity in monolayer graphene produced by mechanical exfoliation using scotch tape [3, 28]. After this invention, many researchers attempted to exfoliate layered 2D materials such as hexagonal boron nitride (h-BN), black Phosphorus (bP), TMDs (MX_2_: MoS_2_, WS_2_, etc.), and more to obtain their monolayers [29–36]. Usually, the micromechanical exfoliation of 2D materials using scotch tape typically yields high-quality flakes of small size which limits its utility for fundamental investigations and is not very appropriate for practical applications. To broaden the applications of 2D materials in practice, large-area thin films of 2D materials with high crystalline quality and spatial uniformity are required. In this context, CVD, a bottom-up synthesis approach was developed which provides the production of continuous films of various 2D materials including graphene, h-BN, and TMDs in a highly scalable manner (Fig. 3a) [37–45]. To synthesize the mono- or few-layer TMDs, precise optimization of growth conditions is necessary as nanosheets are not only structurally unstable but also contain high surface area and energy. Fortunately, the atomic structure of layered MX₂ TMDs is inherently anisotropic, which is advantageous for the formation of their two-dimensional nanosheets. The CVD growth of most TMDs is based on the creation of molybdenum or tungsten films in the presence of sulfur [46–53], selenium [54–57], or tellurium [58, 59] environment. Many transition metals or their oxide precursors have high melting points, making them difficult to be synthesized. For example, Zhan et al. demonstrated the CVD growth of large-area MoS_2_ films through the sulfurization of Mo thin films deposited on SiO_2_ substrate at 750℃ [42] and Najmaei et al. synthesized MoS_2_ on SiO_2_ substrate using MoO_3_ and sulfur as a precursor at 850℃ [38]. To decrease the growth temperature, a molten-salt-assisted method was developed, which enabled the production of ceramic powders at lower temperatures thereby monolayer TMDs were successfully grown at relatively lower temperatures (Fig. 3b) [60, 61]. For example, Zhou et al. synthesized a variety of TMDs, a total of 47 compounds, by using the molten-salt-assisted CVD method [62]. They synthesized not only 32 binary compounds based on the transition metals Ti, Zr, Hf, V, Nb, Ta, Mo, W, Re, Pt, Pd, and Fe but also complex alloys-11 ternary, one quaternary, one quinary and heterostructured compounds. The CVD-grown TMDs have advantages in grain size, with the average grain size ranging from a few tens to hundreds of micrometers [63]. However, CVD has prominent limitations in reliability, uniformity, growth temperature and synthesis area. As illustrated in Fig. 3a, in the CVD growth, the precursor is located inside the chamber, which inhibits precise control over the amount of reacting precursor.

To adopt 2D materials in practical electronic devices, 2D films must achieve wafer-scale uniformity with minimal defects [64, 65]. MOCVD is one of the promising routes to synthesize large-scale 2D films [6, 13, 66–68]. As illustrated in Fig. 3c, it can precisely control the precursor flow by utilizing the vaporizing organometallic compounds that can be supplied into the chamber in the gas phase. Furthermore, the MOCVD method can be used in low-temperature synthesis because most organometallic compounds can be decomposed at low temperatures (250 ~ 300℃) [69–74]. Kang et al. first succeeded in synthesizing MoS_2_ and tungsten disulfide (WS_2_) on 100 mm Si/SiO_2_ wafer with excellent uniformity and high electrical performance (e.g., high mobility) via MOCVD approach (Fig. 3d) [19]. They demonstrated wafer-scale batch fabrication of MoS_2_ using MOCVD which exhibited high carrier mobility of 30 cm^2^ V^−1^ s^−1^ at room temperature and up to 114 cm^2^ V^−1^ s^−1^ at 90 K with 99% device yield. The MOCVD-grown TMDs typically exhibit a much smaller grain size (a few hundred nanometers) in comparison to that of the CVD-grown (a few hundred of micrometers) [63]. The small grain size leads to a high grain boundary density, which reduces the carrier mobility. To enlarge the grain size, several attempts have been made, including periodic ripening, epitaxial growth, single crystal seed growth, and more. Seol et al. developed a novel way to synthesize 150 mm wafer-scale films of monolayer n-type TMDs with larger grain sizes on SiO_2_ substrates within 12 min (Fig. 3e) [75]. To achieve a layer-by-layer growth mode, they promoted vertical Ostwald ripening by interrupting the precursor injection which leads to suppressed secondary nucleation and enhanced lateral growth rate. Wafer-scale batch fabrication of FET arrays was conducted, and the results showed highly uniform and remarkable performance. Electron mobility increased with larger grain sizes, exhibiting 4.9 cm^2^ V⁻^1^ s⁻^1^ for a grain size of 40 nm and 9.4 cm^2^ V⁻^1^ s⁻^1^ for a grain size of 120 nm, respectively. Chubarov et al. developed a new multistep MOCVD growth method to synthesize WS_2_ monolayer on 50 mm c-plane sapphire (Fig. 3f) [76]. They achieved epitaxial growth with minimal in-plane rotation (0.09°) and bilayer coverage (< 1%) by varying the growth temperature and growth rate in a multistep manner. The TEM results show that the grain size is up to the micrometer scale, and photoluminescence spectra at 80 K confirmed the absence of prominent defect states. The fabricated FETs using this TMD film exhibited high performance with electron mobility of 16 cm^2^ V⁻^1^ s⁻^1^ and on/off ratio of ~ 10^7^ which provide the possibility of wafer-scale single-crystal TMD growth via MOCVD approach with a quality close to those of exfoliated flakes.

In addition, various attempts have been made to synthesize large-scale, high-quality n-type TMDs by playing on precursor selection, salt-assisted growth, substrate selection, and more [63, 79–83]. The development of bottom-up CVD or MOCVD processes for TMD production has not been limited to n-type materials. For example, Liu et al. designed a new MOCVD method called the periodical varying-temperature ripening approach, which can be adapted to produce various TMDs including p-type tungsten diselenide (WSe_2_) (Fig. 3g) [77]. To enlarge the grain size, they periodically elevated the growth temperature and reduced the metal precursor to encourage ripening and suppress the nucleation. This method not only enlarged the grain size (up to 20 μm) with reduced nucleation but also improved crystalline quality. Notably, this process is free of impurities because it does not involve any additives or etchants (Fig. 3h). Xu et al. devised a novel method to synthesize wafer-scale single-crystal 2H molybdenum ditelluride (MoTe_2_), a p-type TMD material, on an amorphous substrate [84]. The 2D epitaxy was initiated at an implanted single crystal seed and propagated across the 1-inch wafer with excellent homogeneity. The fabricated transistor arrays with synthesized MoTe_2_ film demonstrated high electrical performance with a hole mobility ~ 45 cm^2^ V⁻^1^ s⁻^1^ and an on/off ratio of ~ 10^4^ with a 100% device yield.

## 2D materials-based CMOS fabrication approaches

**Fig. 4:**
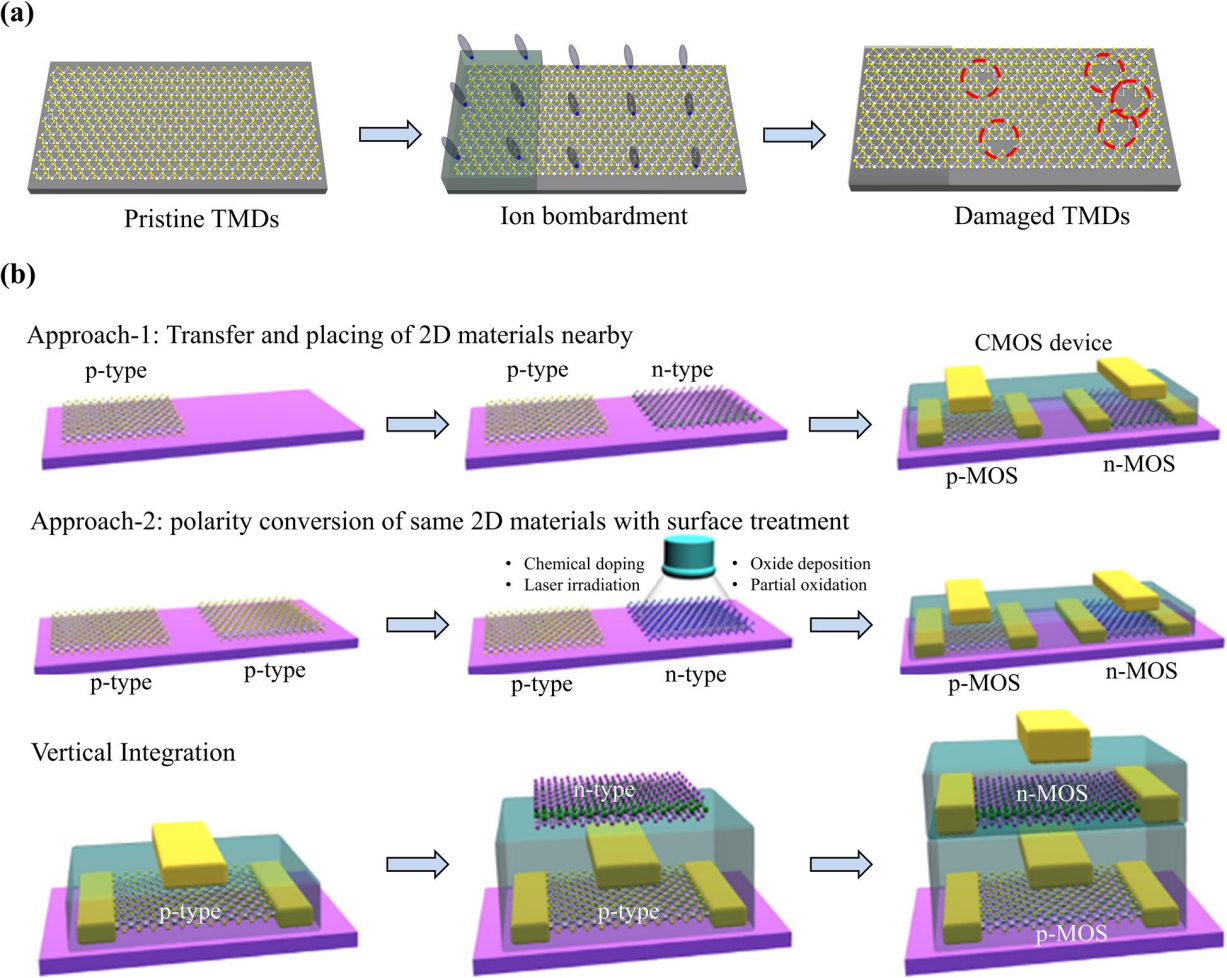
Use of 2D semiconductors in CMOS electronics. **a** Schematics representation showing the challenges associated with the typical ion implantation-based doping which leads to extensive damage in their lattice. **b** The strategies to realize the 2D materials-based CMOS electronics. The integration of two dissimilar 2D semiconductors (p- and n-type) nearby with a selective transfer on the same substrate to fabricate interconnected p- and n-channel FETs is one approach. Alternatively, selective conversion via treating the specific area of the same TMD via different approaches to tune charge carrier polarity type

Ion implantation is a primary technology for doping silicon semiconductors. Dopant ions are implanted into the silicon lattice utilizing high kinetic energy, followed by high-temperature annealing (activation), which enables the creation of a significant number of defects leading to the generation of free charge carriers (both holes and electrons depending on dopant ions) [85–87]. The ion implantation doping in bulk semiconductors like Si allows a precise control of conductivity and polarity tuning that enables the realization of high-performance CMOS integration. Adopting ion implantation doping for 2D materials is challenging because the high-energy ions create significant damage in the atomically thin 2D films [88, 89]. In the ion bombardment process, the collision of ions with high kinetic energy on 2D materials surface generates not only substitutional defects but also pinholes and even tearing of the film (Fig. 4a).

To address the lack of doping methods, researchers have attempted to develop alternative ways to fabricate CMOS devices via transferring the TMD films of opposite polarity nearby on the same substrate. The transfer method, a widely used technique to locate the TMDs at designated positions, causes minimal damage to TMD films, and the process is relatively simple. It relies on peeling the TMDs film off from the growth substrate with a supporting layer and then placing the film on the target substrate [90–93]. Researchers also attempted to deposit TMD crystals in limited areas using pre-patterning, laser-assisted synthesis, and thermodynamically driven selective growth mechanisms [94–97]. Even though the selective growth and transfer techniques offer advantages for fabricating both TMD-based p-MOS and n-MOS on a single substrate, doping is necessary to advance the 2D materials-based electronics. To tune the carrier polarity in TMDs with minimal damage, researchers also attempted to develop alternative methods other than ion implantation, including chemical doping [98–103], oxide deposition [104–107], channel oxidation [102, 108–110], laser irradiation [111] and so on (Fig. 4b) [112–114].

### CMOS devices developed with transfer techniques

**Fig. 5:**
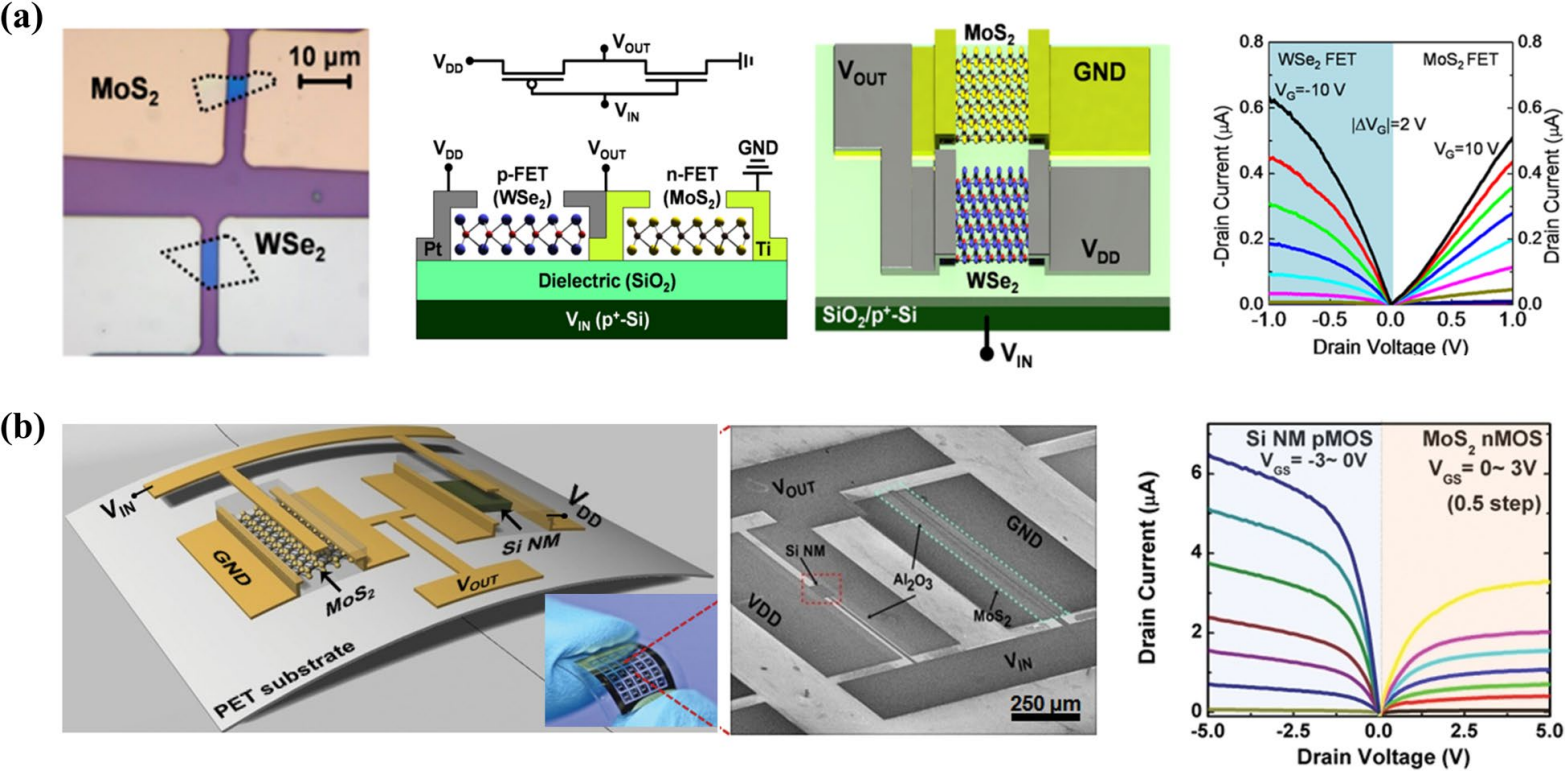
Fabrication of 2D semiconductors-based CMOS devices via transfer-based approach. **a** n-MoS_2_ and a p-type WSe_2_ flakes placed nearby on a SiO_2_/p^+^-Si substrate using mechanical exfoliation and imprint-transfer-based approach. Circuit layout and the schematic diagram of the fabricated CMOS device. The 3D illustration of MoS_2_ and WSe_2_-based hetero-CMOS inverter in which SiO_2_/p^+^-Si worked as a universal gate. The obtained output characteristics of the fabricated device. Reproduced with permission [115]. Copyright 2015, American Chemical Society*.* **b** Schematic illustration of a flexible CMOS complementary inverter developed by integrating CVD MoS_2_-based n-MOS with Si nanomembrane-based p-MOS on a plastic substrate (left). Corresponding photographic (inset) and SEM images (middle) of the developed device unit. The right panel shows the output characteristics, of p-type TFT and n-type TFT obtained with scanning *V*_GS_ = − 3 to 0 V for p-MOS, and 0 to + 3 V for n-MOS. Reproduced with permission [50]. Copyright 2016, Wiley‐VCH GmbH

The CMOS technology typically utilizes both n-type and p-type metal–oxide–semiconductor field-effect transistors (MOSFETs), which work together to create efficient and low-power electronic components for variation functionalities. MoS_2_ or other TMDs usually exhibit n- or p-type electrical characteristics that cannot be modulated in a way similar to that of the conventional ion implantation-based doping approach, which obstructs the use of TMDs in developing CMOS devices. The alternative approach is to assemble the n- and p-MOS fabricated with different TMDs possessing p- and n- electrical characteristics. For example, Guan et al. demonstrated the fabrication of a 2D CMOS inverter exploiting exfoliated MoS_2_ and WSe_2_ as n- and p-type channel materials, respectively (Fig. 5a) [115]. By using the exfoliation and transfer method, they fabricated the n-MOS, p-MOS, and hetero-CMOS inverter also on a single substrate which demonstrated the CMOS digital logic operations (NOT, OR, and AND) with excellent performance. The fabricated CMOS device exhibited a voltage gain of above 20, a small switching delay of ∼800 μs, sub-nanowatt power consumption, and an almost ideal noise margin. In another study, Das et al. transferred CVD-grown large-area MoS_2_ nanosheet near a pre-fabricated silicon nanomembrane (Si NM) p-MOS array to construct n-MOS for CMOS logic circuit integration (Fig. 5b) [50]. The transferred CMOS inverter exhibits air-stable voltage transfer characteristics with a high voltage gain of ~ 16 and sub-nanowatt power consumption. In addition, the flexibility of TMDs and Si NM, along with the transfer method, enables the fabrication of CMOS logic circuits on flexible polymer substrates. CMOS devices on flexible substrates also demonstrated reliable electrical performance under strain. This approach paves the way for highly flexible CMOS devices through 2D materials.

For 2D CMOS technology to mature, an in situ or continuous fabrication process without transfer is required. Selective area synthesis is a promising approach for a continuous fabrication process that grows selective p- or n-type TMDs (or single crystalline TMDs) only in designated or pre-patterned areas. This method allows a transfer-less, industry-compatible TFT fabrication. Kim et al. developed a novel method for synthesizing the single crystalline monolayer and heterostructures of TMDs at selective areas on a 2-inch wafer [96]. They made trench patterns on the SiO_2_ wafer using HfO_2_ to define the micrometer-scale growth pocket and successfully synthesized single crystal TMDs in designated areas of the amorphous substrate. Selectively grown monolayer TMDs exhibited high carrier mobility because each domain is single crystalline, with values of 72.8 cm^2^ V^−1^ s^−1^ for WSe_2_ and 62.2 cm^2^ V^−1^ s^−1^ for MoS_2_, respectively. Furthermore, they were able to synthesize not only monolayers but also single crystalline bilayers and even heterostructures which can be further advanced to fabricate the p-n junctions or CMOS devices. However, this out-of-plane heterostructure is limited to unipolar and p–n junction devices unless monolithic 3D integration is adopted. In this context*,* Zhang et al. demonstrated the synthesis of in-plane heterostructure of monolayer TMDs, using laser patterning combined with a thermal etching process [97]. Firstly, the single crystal WS_2_ was grown via a CVD process followed by laser patterning and thermal etching to create the periodic triangular defects within the CVD-grown WS_2_. After that, CVD with different precursors under optimized conditions was conducted to fill in the etched region of the firstly grown WS_2_ with WSe_2_, enabling the synthesis of in-plane heterostructure. They reported that the endo-epitaxial approach can be used not only for WS_2_/WSe_2_ heterostructure but also for other structures, WS_2_, WSe_2_ for matrix, and WSe_2_, MoS_2_ for the fill. By combining these selective growth methods, it is anticipated to synthesize both single crystalline p-type and n-type TMDs on a single substrate, specifically in designated areas, to fabricate high-performance CMOS logic circuits.

### CMOS devices developed with local polarity conversion

**Fig. 6:**
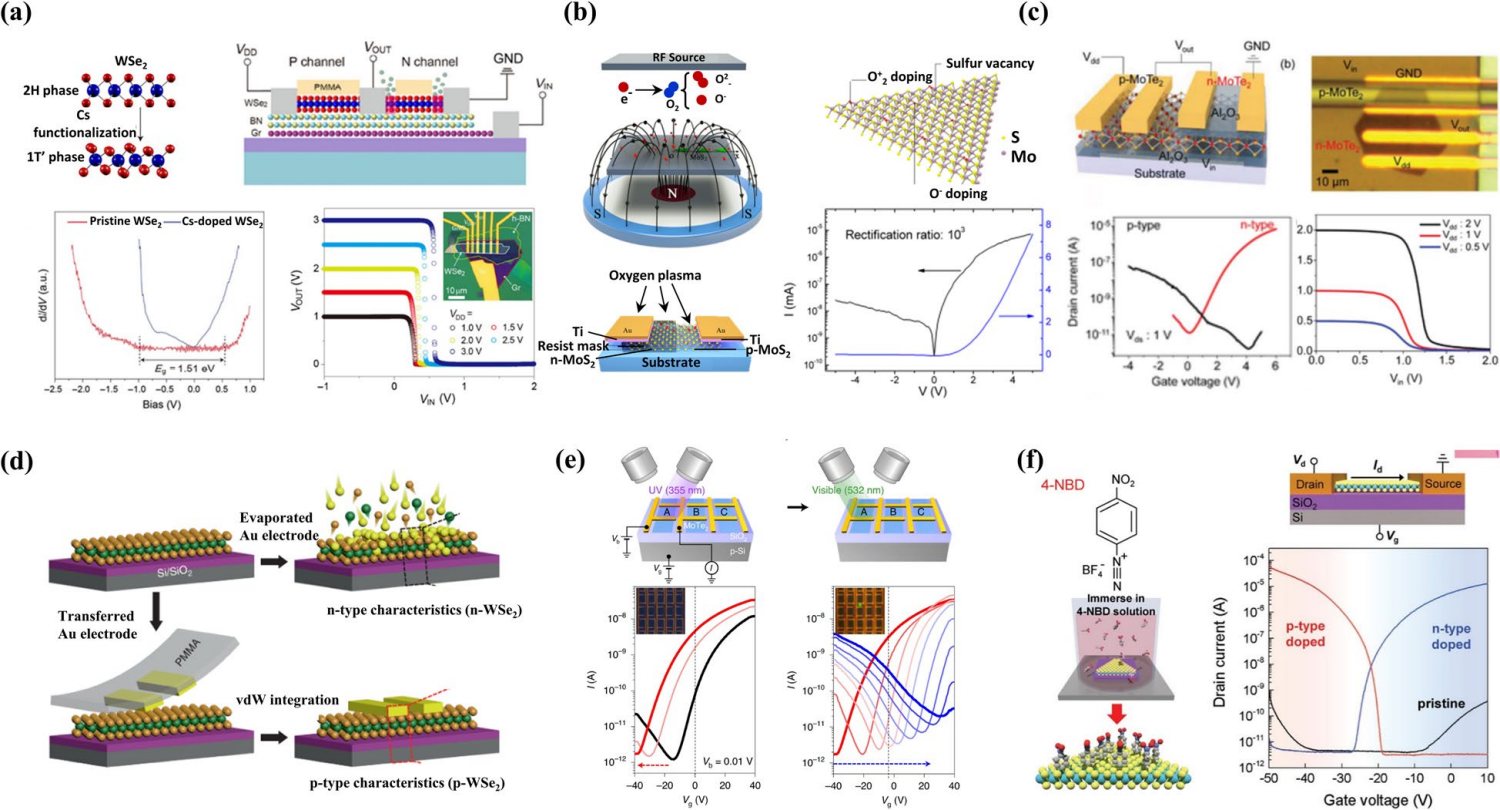
CMOS devices developed by selective polarity conversion of the same TMDs via various surface treatment approaches. **a** Schematic illustrations of the 2H and 1 T- phase WSe_2_ and a WSe_2_ logic inverter. The charge carrier polarity in one WSe_2_ channel was converted to n-type with Cs contact doping. The below plots represent the STS scans of pristine and Cs-modified WSe_2_ including the obtained output characteristic. Reproduced with permission [113]. Copyright 2021, Springer Nature. **b** Schematic representation of the oxygen plasma-based doping approach to dope MoS_2_ crystal (top). Illustration of a lateral p–n junction created with area selective oxygen plasma doping in MoS_2_. The right panel shows the I–V characteristics of the fabricated p-n diode. Reproduced with permission [119]. Copyright 2021, IOP publishing*.* **c** Schematic illustration and optical image of a CMOS inverter fabricated with CVD-grown single MoTe_2_ crystal. The charge carrier polarity in MoTe_2_ was modulated by using an interfacial Al_2_O_3_ layer. Bottom plots represent the transfer characteristics of TFTs fabricated with bare and Al_2_O_3_ coated MoTe_2_. The bottom right plots show the voltage transfer characteristics (VTC) of the fabricated CMOS inverter. Reproduced with permission [59]. Copyright 2019, Willey‐VCH GmbH. **d** Schematic representation of realizing the n- and p-type electrical polarity in mechanically exfoliated WSe_2_ flakes. The thermally evaporated metal onto the WSe_2_ lattice resulted in defects and damage, which provided n-doping effects. In contrast, the FETs fabricated with transferred metal exhibited a clean and sharp interface that resulted in intrinsic p-type electrical characteristics. Reproduced with permission [125]. Copyright 2020, Springer Nature*.* **e** The photo-induced polarity conversion in MoTe_2_. The transfer characteristics of 2H-MoTe_2_ back-gated FETs under the exposure of the UV laser (*λ* = 355 nm, left) and a visible laser (*λ* = 532 nm, right). The transfer characteristics exhibited n-type doping for red and p-type doping for blue light exposure. Reproduced with permission [111]. Copyright 2020, Springer Nature. **f** The chemical treatment-based doping in WSe_2_ crystals with 4-NBD for p-type and DEA molecules for n-doping. The corresponding FET structure and obtained electrical characteristics in the fabricated devices. Reproduced with permission [114]. Copyright 2019, Willey‐VCH Verlag GmbH

Point defects in TMDs, particularly chalcogen vacancies, result in innate n-doping of the TMDs so the fabrication of n-type TFT is widely studied [116–118]. However, the innate n-doping presents a significant obstacle in fabricating the p-type TFTs. To overcome this issue, researchers attempted to p-dope the innate n-type TMDs in several ways such as metal substitution, chemical doping, contact engineering, selective oxidation, and so on [100–103]. To convert p-type WSe_2_ into n-type, Zheng et al. used the cesium (Cs) doping method near the contact region, making ideal n-type WSe_2_ TFT with Ohmic contact and minimal contact resistance (Fig. 6a) [113]. Electron doping from Cs induces a phase transition from semiconductor (2H) to metal (1 T’), which can be used as a contact electrode with low contact resistance. The 2H-1 T’ hetero-phase contact enables the Ohmic contact with a low Schottky barrier of 19 meV and contact resistance of 0.9 kΩ·μm. By adopting this method, they attained high switching performance, subthreshold-swing (SS) of 61 mV/dec, on/off ratio 10^9^ with high electron mobility up to 68 cm^2^ V^−1^ s^−1^. Additionally, by using this hetero-phase WSe_2_, they fabricated the inverter which exhibited a power consumption of 28 pW and a voltage gain of 270. The Cs doping method paves the way for easily achieving n-type WSe_2_ TFT and CMOS logic devices. Ji et al. investigated a chemical doping method that can be used for both n-type and p-type doping, not only to increase carrier mobility but also to tune the polarity of CVD-grown monolayer WSe_2_ [114]. They immersed the WSe_2_ in two different aqueous solutions of 4-nitrobenzenediazonium tetrafluoroborate (4-NBD) and diethylenetriamine (DETA) for p-doping and n-doping of the WSe_2_, respectively. Spectroscopic measurements and DFT calculations support the experimentally obtained results indicating that each chemical facilitates charge transfer to the WSe_2_ film, leading to an increase in carrier mobility and even polarity inversion (82 cm^2^ V^−1^ s^−1^ for holes and 25 cm^2^ V^−1^ s^−1^ for electrons). Using this method, they demonstrated a CMOS inverter exhibiting a voltage gain of 10 with an extremely low power consumption of 0.17 nW. Furthermore, they configured the p–n junction within a single WSe_2_ crystal showing prominent rectification and photoresponse. Wu et al. reported the polarity conversion of n-type MoS_2_ into p-type using oxygen plasma doping with a toroidal-magnetic-field (TMF) to fabricate the p-MOS based on MoS_2_ (Fig. 6b) [119]. By restricting the energy of oxygen ions, a rapid oxygen substitution rate with negligible lattice defects was confirmed through Raman-, PL-spectroscopy, and XPS measurements. Effective doping with low lattice defects resulted in the high performance of p-type MoS_2_ FET, achieving an on/off ratio of 10^7^, hole mobility of 115.2 cm^2^ V^−1^ s^−1^, and a SS of 137 mV dec^−1^ at room temperature. However, these methods generate defects in the channel region, which might hinder the reliability of the process and long-term stability of devices.

Atomic layer deposition (ALD) is a widely used method for forming the gate oxide in the TFT process*.* However, during the ALD process, the channel material can be n-doped [105, 106], or the ALD-grown gate oxide itself might occur n-doping due to oxygen vacancies in the oxide [104]*.* Park et al. effectively utilized the ALD-induced doping effect to tune the polarity of TMDs from p-type to n-type (Fig. 6c) [59]. They selectively deposited an ultrathin Al_2_O_3_ layer on the CVD-grown MoTe_2_ and achieved the polarity change in a nondestructive manner. The low operating temperature during the gas-phase reaction in ALD chamber lead to the damage-free doping of MoTe_2_. The electron doping effect after the ALD process was confirmed by observing the shift in Fermi level and work function using XPS and UPS analysis. N-doped MoTe_2_ exhibited high electron mobility of 8.9 cm^2^ V^−1^ s^−1^ along with a low Schottky barrier height of 28.4 eV. Furthermore, they selectively doped the MoTe_2_ and configured the CMOS inverter on a single MoTe_2_ crystal, which showed a voltage gain of 9.2 with a high modulation frequency of 1 kHz. Contact metal engineering is an important factor that strongly affect the TFT performance. The relative position of the work function of the contact metal and channel materials determines not only the contact properties such as contact resistance, Ohmic contact, or Schottky contact—but also the polarity of TFT [120–123]. Furthermore, since TMDs are atomically thin materials metallization methods also affect the characteristics of TFTs due to damage occurring during the contact process [124]. Kong et al. devised a doping-free approach to tune the polarity of WSe_2_ by simply changing the metallization method while using the same contact metal (Fig. 6d) [125]. Unlike conventional Au deposition, the integration of low-energy van der Waals (vdW) contact electrodes exhibits p-type behavior. They combined both conventional Au deposition and vdW integration methods to fabricate the doping-free CMOS logic devices. The inverter exhibited a high voltage gain of 340 at V_dd_ of 5.5 V and other circuits such as NAND and NOR were also demonstrated. Reconfigurable and selective doping of semiconductors is essential for fabricating complex and advanced integrated circuits. Ion implantation is an effective way to dope the bulk semiconductor and is widely used in VLSI applications. However as mentioned earlier, ion implantation is not applicable to 2D materials technology regarding defects generation. In this scenario, Seo et al. reported a novel reconfigurable doping method to convert the polarity of the TMDs without chemical doping or charge transfer layer (Fig. 6e) [111]. The polarity of MoTe_2_ and WSe_2_ were changed from p-type to n-type and vice versa, by illuminating them with the different wavelengths of the laser. The mechanism of photo-induced doping involves ultraviolet laser light promoting the formation of Te self-interstitial defects, while visible light creates the O_Te_ and O_Mo_ point defects in the MoTe_2_ lattice. This method is quite stable because it promotes substitutional defects rather than vacancies or interstitial defects, which are less stable than substitutional defects due to the absence of strong covalent bonding. Thus, when we encapsulate the channel with h-BN, it remains stable under ambient exposure for several months. This work paves the way for the development of 2D materials for M3D applications because it can replace the ion bombardment process used in the Si CMOS process. In another work, Ji et al. demonstrated to control the electrical polarity in monolayer WSe_2_ by utilizing the facile approach of chemical doping. They exploited 4-nitrobenzenediazonium tetrafluoroborate and diethylenetriamine to convert ambipolar WSe_2_ field-effect transistors to p- and n-type, respectively (Fig. 6f) [126].

## 2D materials-based flexible CMOS devices

ICs are essential components that govern data processing, transmission, and power management while simultaneously providing communication between external units [127]. A fully functional IC consists of several electronic units such as resistors, capacitors, diodes, and particularly the CMOS components including p- and n-type transistors which are integrated in a particular manner to perform specific tasks, such as logic operations and memory functions. These various components of an integrated circuit work together to provide complex functionalities in a compact form, enabling a wide range of applications in electronics. 2D materials are not only utilized in the development of CMOS devices on rigid platforms, but their exceptional mechanical merits make them highly auspicious candidates for developing electronic devices on flexible platforms with a variety of promising applications in wearable healthcare electronics. Due to the limitation in the overall thickness of the device to maintain flexibility, wearable/stretchable electronic devices need to have high-performance and power-efficient units [128, 129]. Conventional ICs composed of rigid Si CMOS components are not very appropriate for flexible applications [130]. The exceptional superiority of 2D materials in electrical characteristics as well as mechanical flexibility in their ultrathin form makes them highly promising alternatives that are potentially capable of revolutionizing the arena of high-performance flexible ICs with multifunctional applications. As a result, numerous 2D materials, including graphene, various TMDs, and black phosphorus, have been investigated for the development of flexible ICs that offer excellent mechanical flexibility and enhanced electrical performance [131].

As advancements in 2D material production continue, flexible ICs based on these materials are evolving continuously. At the early stage of development, mechanically exfoliated 2D materials have been exploited to demonstrate the fundamental electronic units such as FETs on rigid SiO_2_/Si substrates and flexible polymeric platforms [4, 132–138]. Later on, complex electronics operations such as logic functions, inverters, and radio frequency (RF) electronics were demonstrated based on the exfoliated TMDs [18, 139, 140]. Mechanically exfoliated 2D semiconductors typically possess high-quality defect-free single layers that provide improved electrical characteristics such as high charge carrier mobility in a power-efficient manner [141]. Exploiting the unique advantages of defect-free exfoliated 2D materials, Cheng et al. demonstrated the development of flexible RF circuits on polyimide substrate with exfoliated few-layer MoS_2_ flakes [139]. The fabricated multiple MoS_2_ transistor-based logic inverter and RF amplifiers exhibited high-frequency response with low noise and provided a voltage gain up to the gigahertz regime. An RF amplifier is a vital unit of the integrated circuit that increases and manages the signal power for high-quality signal transmission, reception, or processing. The MoS_2_ FET utilized in the RF amplifier exhibited intrinsic cut-off and maximum oscillation frequencies of 13.5 GHz and 10.5 GHz, respectively. In addition, the fabricated RF amplifier delivers a relative voltage gain of ~ 2 in response to a sinusoidal input of up to 300 MHz frequency. An amplitude modulator (AM) is another important electronic unit of RF electronics that retrieves the information encoded in signals modulated onto an RF carrier. Zhu et al. exploited the ambipolar electronic characteristics and high charge carrier mobilities in exfoliated bP-based FETs and developed a flexible amplitude-modulated demodulator on polyimide substrates [140]. The bottom-gated exfoliated black phosphorous-based ambipolar FETs were fabricated on a flexible polyimide substrate and encapsulated with Al_2_O_3_ for increasing air stability. The FETs provided charge carrier mobility of as high as 310 cm^2^ V^−1^ s^−1^ and ~ 3 orders of current modulation with the electric field. The developed exfoliated bP-based transistors on the flexible PI platform possess excellent mechanical robustness that can sustain the uniaxial tensile strain up to 2% and exhibited uniform electric performance up to 5000 bending cycles.

**Fig. 7:**
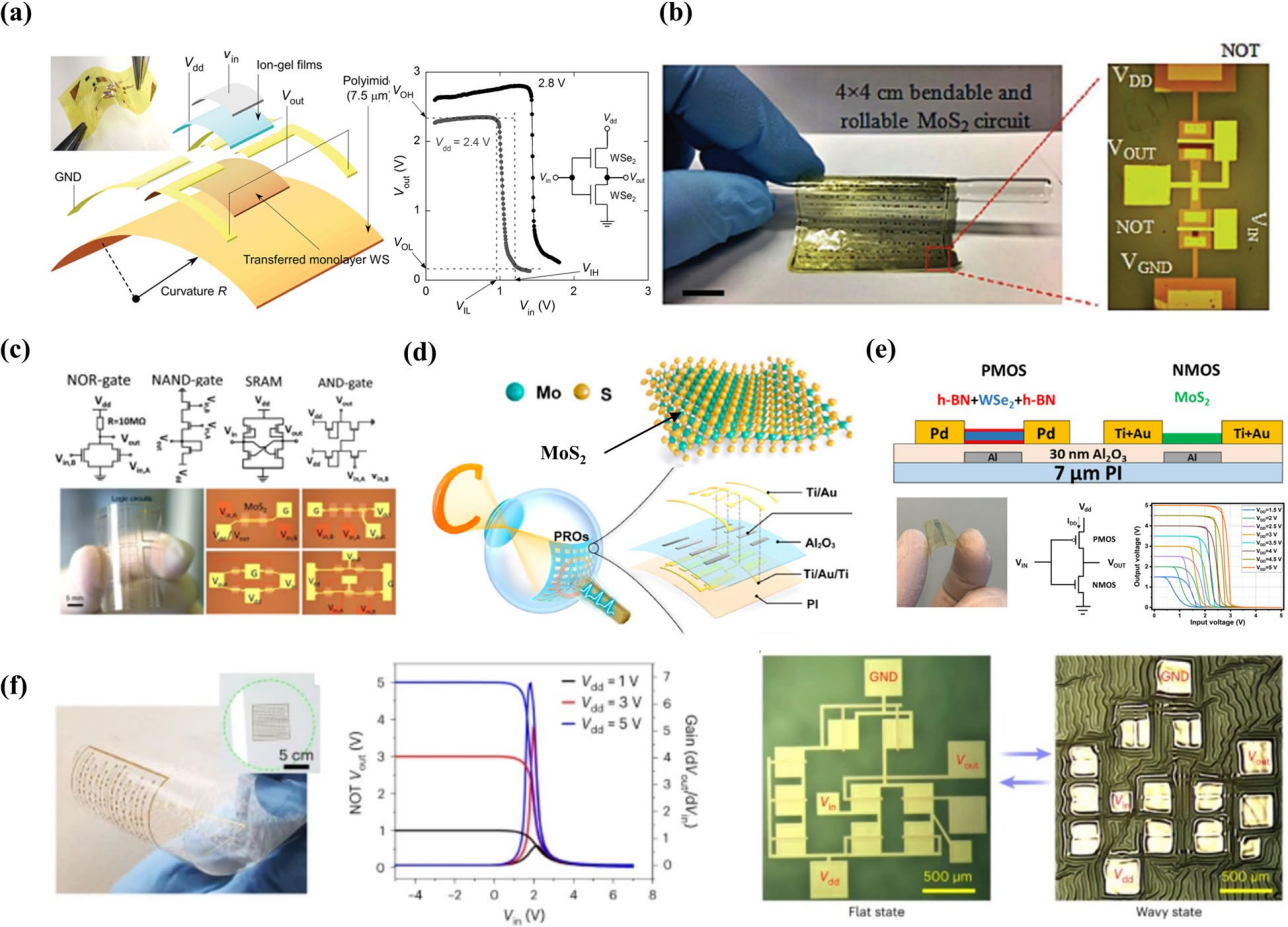
2D materials-based flexible CMOS devices. **a** Schematic representation of a flexible inverter fabricated with transferred MoS_2_ and monolayer films. Reproduced with permission [18]. Copyright 2016, Willey‐VCH Verlag GmbH. **b** Photograph of a rollable logic circuit fabricated with MOCVD grown large area MoS_2_ films. The enlarged view represents the NOT and NAND logic. Reproduced with permission [92]. Copyright 2018, Willey‐VCH Verlag GmbH. **c** The circuit layout and digital photographs of various integrated multistage circuits such as inverters, NOR gates, NAND gates, SRAMs, AND gates, and five-stage ring oscillators. These logic circuits were developed with large area MoS_2_ grown by the MOCVD approach. Reproduced with permission [15]. Copyright 2020, Springer Nature. **d** The circuit layout and a photographic image of a flexible MoS_2_-based artificial retina mounted on a contact lens. The schematic representation presented at left shows the device structure of a PRO developed with large area MoS_2_. Reproduced with permission [144]. Copyright 2023, American Chemical Society. **e** Schematic representation of a CMOS inverter consisting of n-type MoS_2_ and p-type WSe_2_-FETs on flexible substrates. The large area MoS_2_ and WSe_2_ was synthesized by CVD and MOCVD approaches acting n-type and p-type channel materials, respectively. The digital photograph and the obtained electrical characteristics of the fabricated device. Reproduced with permission [145]. Copyright 2024, Willey‐VCH Verlag GmbH. **f** Photograph of a bent logic device developed with MOCVD grown large area MoS_2_ film. The voltage transfer- and gain-characteristics of a NOT logic device operated under different *V*_dd_. The photographs presented right side represent the repeated flexibility in the fabricated logic devices. Reproduced with permission [71]. Copyright 2023, Springer Nature

The ultralow thickness of TMDs-based 2D semiconductors in their monolayer form offered the realization of the ultimately thin active channel in the fabricated FETs. It eventually delivered a more effective gate control and enabled the attainment of an on/off current ratio of more than 10^6^ and a subthreshold swing (SS) as low as 60 mV dec^–1^ [134–136]. The high on/off current ratio and small SS are essential for the effective function of several electronic components such as CMOS inverters and determine the off-state voltage and voltage gain. Therefore, the electrical characteristics of 2D TMDs-based TFTs coupled with their mechanical merits provided a revolutionary stage for the development of low-power logic circuits on flexible platforms. Tosun et al. demonstrated the fabrication of high-gain inverters on rigid substrates with exfoliated WSe_2_ flakes-based CMOS inverters by fabricating p- and n-channel FETs on the same WSe_2_ flake by locally converting the carrier polarity with potassium doping [138]. In another study, Jeon et al. demonstrated a hetero-CMOS inverter by utilizing the p- and n-channel bottom-gated FETs fabricated on WSe_2_ and MoS_2_ flakes exfoliated separately and placed nearby on a glass substrate [115]. Exfoliated TMDs have successfully shown their potential in several CMOS devices for various functions with low power consumption. However, the typical size of these devices is less than a few µm^2^, and only individual units can be fabricated, making them unsuitable for array-based scalable integration in functional flexible electronics. With advancements in the production of large-area, high-quality 2D TMDs using CVD and MOCVD methods, it has become possible to fabricate high-performance ultrathin CMOS devices over large areas on flexible substrates. For example, Pu et al. demonstrated the fabrication of flexible CMOS inverters using a CVD-grown large-area TMD monolayer. They used ion gels as gate dielectric and combined p-type WSe_2_ and n-type MoS_2_ electric double-layer transistors (EDLTs) to validate logic operations in a CMOS device fabricated on the flexible PI substrate (Fig. 7a). The fabricated flexible inverters exhibited high voltage gain (>100), with negligible off-state voltage, large total noise margin (~ 62%), low power consumption, and reasonably good switching speed (> 1 kHz). The developed large-area inverters onto flexible plastic substrates exhibited mechanical robustness and maintained stable operation under the repeated bending of the device up to a curvature radius of 0.5 mm.

Because it is difficult to grow 2D semiconductors directly on flexible polymer substrates due to their low-temperature tolerance, 2D materials-based large-area flexible devices were typically realized by first synthesizing 2D materials on rigid SiO_2_/Si or sapphire substrates using CVD or MOCVD approaches, which are then transferred to the desired polymer substrates for fabrication. This transfer step in the development of 2D materials-based flexible electronics is crucial, as the hazardous acid-based chemical etching typically utilized to release the 2D materials from the growth substrates which substantially degrades the material quality and thereby the device performance. In this context, Shinde et al. developed surface energy-assisted water-based processes to transfer the MOCVD-grown TMDs and demonstrated the fabrication of an n-MOS inverter [92]. The surface of the SiO_2_/Si growth substrate was treated with diluted hydro fluoric (HF) acid before inserting the substrate in the growth chamber. The interaction of the top SiO_2_ surface of the substrate with HF enhances the strongly adhering silanol groups at the MoS_2_/SiO_2_ interface that allows the damage and degradation-free transfer of large-area MoS_2_ films in the water without requiring any acid-based releasing agent. They demonstrated the fabrication of inverter, NAND, and NOR gates directly on the flexible PI substrate via transferring the MOCVD-grown MoS_2_ via a water-assisted transfer approach (Fig. 7b). To realize the n-MOS inverter, the MoS_2_-based two FETs were organized in a way where one transistor acted as a switch while the other acted as an active load. The inverter function was realized by applying the supply voltage (V_DD_) at the drain of the load transistor and the input voltage (V_IN_) at the gate of the switch transistor. Using the developed water-assisted wet transfer method, the combination of several logic operations such as AND, OR, and XNOR was demonstrated on the flexible platform. The fabricated flexible logic circuits exhibited excellent mechanical robustness without any noticeable performance degradation with repeated bending up to a bending radius of 3.2 mm.

The advancement in the CVD and MOCVD synthesis techniques enabled the production of high-quality 2D materials with uniform thickness over large areas. Different 2D materials obtained through these approaches have been used to showcase a range of large-area flexible electronic devices, featuring numerous electronic components that exhibit excellent performance uniformity and consistency [142, 143]. For example, Li et al. reported the fabrication of flexible logic circuits over a large area using wafer-scale (100 mm) monolayer MoS_2_ films synthesized via a modified CVD approach (Fig. 7c) [15]. The high-density FETs (1,518 transistors/cm^2^) were fabricated on large area flexible polyethylene terephthalate (PET) substrate that exhibited on/off current ratio, on current density and charge carrier mobility of as high as ~ 10^10^, ~ 35 μA μm^−1^ and ~ 55 cm^2^ V^−1^ s^−1^, respectively. These high-performance FETs were integrated in a particular manner to create flexible integrated circuits performing the operations of various logic circuits such as inverters, logic gates (NOR, NAND, AND), static random access memories, and five-stage ring oscillators. The fabricated logic circuits on PET substrate exhibited a stable performance even when the devices were subjected to the bending-induced strain of 1% up to 1000 cycles of repeated bending. In another study same research group demonstrated the development of flexible photo-responsive ring oscillators (PROs) using CVD-grown MoS_2_ monolayers intended to replicate the functionality of the human eye (Fig. 7d) [144]. In each PRO, the monolayer MoS_2_-based FETs were organized in a manner to create a three-stage ring oscillator comprising three inverters connected in series along with an extra inverter connected to an output buffer. The array of ring oscillators was fabricated on flexible and biocompatible substrates as vision pixels in a manner to mimic the human retina as an artificial visual system for image recognition and memorization. The developed PRO units exhibited stable and reliable functionality even when the device was subjected to mechanical strain under bending. The fabricated PROs-based artificial vision system needed ultralow-power of 12.4 nW/pixel which enabled to power the artificial retina wirelessly via an RF coil. Recently, Piacentini et al. demonstrated the fabrication of both n-MOS and p-MOS with n-type MoS_2_ and p-type WSe_2_ on a flexible substrate (Fig. 7e). The large area MoS_2_ and WSe_2_ were synthesized by CVD and MOCVD approaches which acted as n-type and p-type channel materials, respectively.

The fabrication of 2D materials-based flexible electronic circuits typically relied on the synthesis of high-quality active materials at an elevated temperature on a rigid growth substrate followed by their transfer to a desired flexible substrate for device fabrication. Due to the low thermal tolerance of the flexible polymeric substrates, the synthesis of 2D materials directly on such substrates remains challenging. Although this growth and transfer method is the most widely used for developing flexible applications based on 2D materials, it has the drawback of causing degradation in the electrical properties of the active 2D materials during the transfer. To this end, in a recent work, Hoang et al. reported the development of a strategy for the synthesis of TMDs, particularly MoS_2_, directly on the flexible polymeric substrates at low temperature (150 ºC) via the MOCVD technique and demonstrated the fabrication of MoS_2_ based logic circuits on ultrathin glass (UTG) and polymer substrates (Fig. 7f) [71]. The functional integrated circuit fabricated on flexible and stretchable substrate consisted of 5-stage ring oscillators and differential amplifiers that offered the oscillation frequency of 120 kHz at a *V*_dd_ of 10 V. The low-temperature synthesis enabled the fabrication of devices directly on flexible substrates, without additional transfer processes which avoided the contamination and damage-induced material quality degradation. Consequently, the fabricated devices exhibited excellent mechanical robustness under repeated stretching and shrinking and showed performance similar to that of the device fabricated on rigid substrates.

## 2D material-based M3D devices

As conventional semiconductor-based CMOS electronics approaching the limit of Moore’s law in increasing the integration density, the search for alternative materials is necessary. To maintain the device miniaturization trend, traditionally shortening channels have been attempted, however, the appearance of short channel effects imposes challenges. To address this challenge, modifications in the device structure have been made and FinFETs and GAAFETs structures have been developed with conventional bulk semiconductors [146–149]. Even though substantial success has been achieved by utilizing these geometries, further miniaturization is challenging due to the scattering-induced degradation in carrier mobility. In this context, the vertical integration of various active layers has been attempted which gained substantial success in further advancing the integration density. The through-silicon-via (TSV)-based 3D integration has already been commercialized in which all tiers are fabricated parallelly followed by stacking them by a bonding process [150, 151]. Monolithic-3D (M3D) integration is another form of 3D integration, in which different device tiers including active layers, back end of line** (**BEOL) layer, and dielectric interlayer etc. are consecutively fabricated on the same wafer [152, 153]. Even though 3D integration has been well employed in the contemporary silicon CMOS industry, the essential electronic units in M3D integration are typically confined to the surface of the silicon substrate, which cannot be arranged into multi-layers. 3D integration using face-to-face bonding requires highly accurate and precise alignment of chip-to-chip connections, which presents significant challenges [154]. Additionally, the process temperature for the upper tier manufacturing is limited to a maximum of 500 ºC, exceeding that can damage the BEOL and other constituent layers with substantial deterioration in the performance of the underlying electronic units. To ensure the effective operation of the electronic components in the upper tier, high-temperature processes such as crystallization and dopant activation are generally required during various fabrication steps. These limitations pose several challenges for current Si-based CMOS technology in terms of cost-effectiveness and performance, prompting the search for alternative materials and manufacturing deeds.

**Fig. 8:**
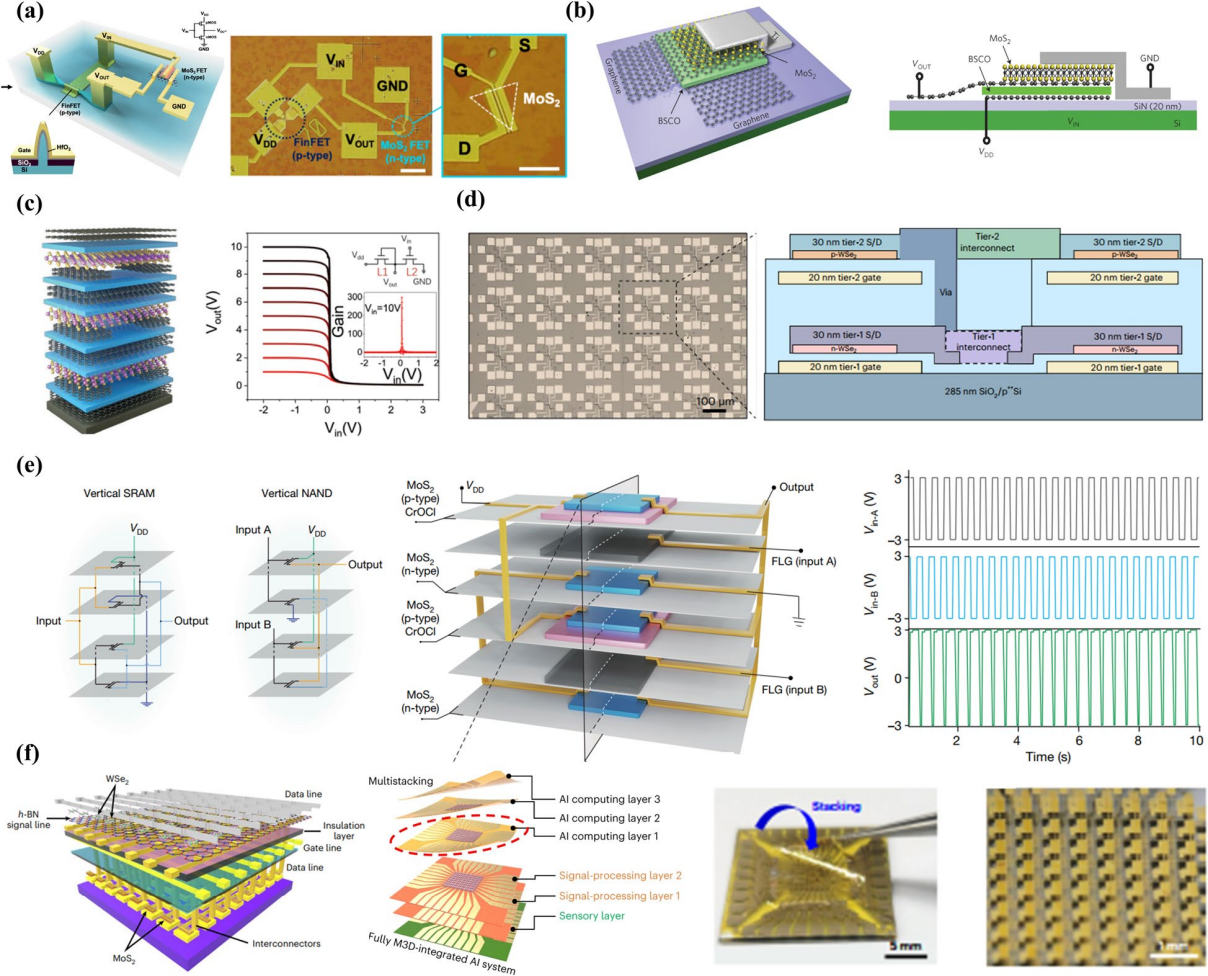
Vertically integrated CMOS electronics. **a** Schematic illustration of the vertical integration of a monolayer MoS_2_ FET on top of p-channel Si FinFET for realization of a CMOS inverter (left panel). The right panel shows the optical image of the fabricated 3D CMOS inverter. Reproduced with permission [161]. Copyright 2023, Springer Nature. **b** A 3D schematic and corresponding cross-sectional device layout of a vertically integrated graphene and MoS_2_-based electronics. Reproduced with permission [164]. Copyright 2012, Springer Nature. **c** Schematic representation of the vertically integrated three-layer FETs organized by layer-by-layer stacking of graphene MoS_2_ and h-BN. The used MoS_2_ crystals were grown by the CVD approach, whereas graphene and h-BN were exfoliated mechanically. The right panel shows the obtained electrical characteristics. Reproduced with permission [162]. Copyright 2020*,* Wiley‐VCH GmbH. **d** Microscopic image and corresponding schematic representation of a two-tier CMOS circuit monolithically integrated with p- and n-channel WSe_2_ FETs. Reproduced with permission [26]. Copyright 2024, Springer Nature. **e** Schematic representation of the vertically integrated 2D materials-based 3D logic electronic including SRAM and NAND gate. The right panel shows the dynamic NAND performance of the fabricated device. Reproduced with permission [12]. Copyright 2024, Springer Nature. **f** Device layout of a M3D-integrated AI processor designed with combining WSe_2_/h-BN-based memristors and MoS_2_-based FETs. The right panels show the complete device and a high-resolution image of a vertically stacked device. Reproduced with permission [13]. Copyright 2023, Springer Nature

The atomically thin 2D TMDs with dangling bonds-free surfaces emerge as alternative materials to conventional bulk semiconductors, that can offer the ultimate limit of miniaturization with the potential to design devices in the vertical direction. 2D materials, which are atomically clean and stable, can be easily stacked into multilayers through artificial assembly to create vdW heterostructures. This approach of physical stacking offers a promising opportunity to advance 3D electronics while addressing the limitations associated with traditional bulk semiconductors in vertical integration. The facile transfer and staking of CVD and MOCVD grown large-area 2D materials at ambient temperature provide a very promising pathway for the development of 2D active channel-based M3D electronics, allowing for high-volume fabrication while adhering to a low thermal budget during processing. This approach effectively addresses additional challenges found in conventional CMOS, such as inter-tier signal delays, and chip overheating issues. The family of 2D semiconductors provides a diverse array of options with controllable band gaps at ultra-low thickness at the atomic scale. Several 2D vdW materials, such as graphene and various TMDs (such as MoS_2_ and WSe_2_) have been explored for the development of M3D electronics with high-density vertical integration [155, 156]. Due to its superior electrical and mechanical characteristics, along with its well-established large-area production, molybdenum disulfide (MoS_2_) is one of the most explored TMDs in the fabrication of low-dimensional electronic devices [12, 13, 22–27, 141, 157–160]. By merging the benefits of 2D materials with existing Si-based CMOS technology, numerous studies have been made toward the vertical integration of 2D TMDs onto Si CMOS for various functional applications [10, 161]. For example, Guan et al. demonstrated the formation of a complementary inverter by integrating a CVD grown MoS_2_ based n-channel FET on a Si p-FinFET having 20 nm fin width via an M3D integration approach (Fig. 8a). The M3D device fabrication was conducted with manufacturing techniques typically employed in the semiconductor industry ensuring compatibility with the standard CMOS processing. The MoS_2_-based n-channel FET was fabricated using Al_2_O_3_ as gate dielectric deposited via e-beam evaporation technique at low-temperature to avoid damage in underlying Si devices. The fabricated CMOS inverter exhibited a symmetrical transfer characteristic with a voltage gain of as high as ~ 38. These demonstrations of 2D materials integration with Si-based devices pave the way for the development of high-density electronics and promote the continued advancement of 3D electronics. However, the integration of 2D semiconductor-based electronics on conventional Si CMOS circuitry using conventional TSV-based manufacturing possesses some concerns of parasitic capacitance and a substantial residual thermal/mechanical stress in device components, which leads difficulties in vertical 3D integration of 2D materials on Si CMOS ICs.

The monolithic 3D integration of all 2D material-based ICs (M3D ICs) represents a promising alternative that effectively tackles the challenges associated with hybrid 2D-3D integration technology. For example, Yu et al. reported the fabrication of vertical multi-heterostructures of layered materials such as graphene, MoS_2_, and cobaltites Bi_2_Sr_2_Co_2_O_8_ and demonstrated high-current–density vertical field effect transistors (VFETs) (Fig. 8b) [161]. The exfoliated multilayer MoS_2_-based n-channel VFET exhibited an on–off ratio of > 10^3^ at room temperature. They also demonstrated the fabrication of a complementary inverter by vertically stacking various layered 2D materials such as graphene, Bi_2_Sr_2_Co_2_O_8_ (p-channel), and MoS_2_ (n-channel) sequentially. In another study, Sachid et al. demonstrate the monolithic 3D integration of n- and p-channel MOSFETs fabricated with exfoliated few layers MoS_2_ and WSe_2_, respectively to develop CMOS electronics [22]. The sequentially fabricated MoS_2_- and WSe_2_-FETs exhibited the electron and hole mobilities of 38 and 238 cm^2^ V^–1^ s^–1^, respectively, which were vertically organized to demonstrate the digital and analog circuits such as inverters, NAND and NOR logics, differential-, common source-amplifier, and signal mixers. The vertically integrated 2D semiconductors-based CMOS inverter exhibited effective operation with a supply voltage (*V*_DD_) as low as 150 mV and exhibited a voltage gain of ~ 10 V/V at *V*_DD_ = 3 V. Tang et al. further advanced the number of tiers in all 2D-based M3D integration and demonstrated a 3 layer device consisting of three typical 2D materials, monolayer MoS_2_, h-BN, and few-layer graphene as semiconducting, dielectric, and contact materials, respectively (Fig. 8c)[162]. The monolayer MoS_2_ triangles were grown by CVD technique, whereas, few-layer h-BN and graphene were exfoliated from the bulk h-BN and graphite crystals, respectively. The fabricated all-2D FETs exhibited excellent electrical performance with ultralow off-current of ~ 100 fA, ultrahigh on/ off ratio ~ 10^10^, subthreshold swing value close to 100 mV dec^−1^, and charge carrier mobility as high as 52 cm^2^ V^−1^ s^−1^. The different layers of vertically integrated all 2D materials-based device exhibited different tasks such as memory, logic, and sensing functions.

Jayachandran et al. demonstrated the wafer scale M3D integration of MoS_2_ and WSe_2_-based n- and p-channel FET arrays with the integration density as high as 10,000 in one tier [25]. The fabricated 2D semiconductors-based 3D circuits exhibited multifunctional capabilities such as sensing, computing, and storage across different tiers. The MOCVD-grown MoS_2_ and WSe_2_ monolayers were first synthesized on 2-inch c-plane sapphire wafers followed by poly-methyl-methacrylate (PMMA) assisted transfer for the vertical integration. The complete device fabrication was conducted at a temperature lower than 180 °C which provided the staking of multiple tiers without creating any damage or degradation to the performance of the underlying tiers. In another study, the same research group reported the M3D integration of WSe_2_ FETs-based complementary circuits over wafer scale, in which both of the n- and p-type FETs were realized with MOCVD-grown monolayer WSe_2_ (Fig. 8d) [26]. The vertically staked M3D devices were fabricated via transferring of WSe_2_ film from the c-sapphire growth substrate to the SiO_2_/Si target substrate via a PMMA-assisted wet transfer approach. The two-tier structure was fabricated consisting of 340 n-type FETs on tier 1 and 340 p-type FETs on tier 2. The inter-tier electronics were coupled together similar to the state-of-the-art packaging with creating densely packed inter-tier vias (300 nm width and 1 µm pitch) [163]. The as-grown WSe_2_ intrinsically exhibited p-type transfer characteristics with Pd contact, whereas n-type characteristics with the same WSe_2_ film were achieved by using the different metal electrode, e.g., Ni as well as exploiting the surface charge transfer doping via depositing ALD Al_2_O_3_. The 3D CMOS circuit fabricated with utilizing p- and n-type electrical characteristics of WSe_2_ demonstrated the successful operations of inverters, NAND, and NOR logics. Importantly, all the steps of device fabrication were performed at a temperature below 200 °C, providing compatibility of integration with BEOL for hybrid 2D/Si CMOS technologies. In a recent study, Guo et al. demonstrated the fabrication of MoS_2_-based vertically integrated CMOS logic circuits (Fig. 8e) [12]. The charge carrier polarity modulation from n- to p-type in mechanically exfoliated few layers MoS_2_ was realized via integrating it on top of a vdW antiferromagnetic insulator chromium oxychloride (CrOCl) through strong vdW interfacial coupling. The band alignment tuning at the MoS_2_/CrOCl interface resulted a hole mobilities and on/off ratios up to as high as 425 cm^2^ V^−1^ s^−1^ and 10^6^ at room temperature, respectively, and fabricated devices exhibited an air-stable electrical performance for more than one year. Exploiting the n-type electrical characteristics in pristine MoS_2_ and p-type characteristics of the MoS_2_ transferred onto exfoliated CrOCl layer, a multi-tier complementary logic circuits were constructed that consists of vertical-SRAMs, -NANDs and -inverters having 14, 6 and 14 vdW layers respectively.

The true potential of 2D materials lies in their capability to incorporate flexibility into the devices which is hard to achieve with the conventional bulk semiconductors. Kang et al. reported the monolithic 3D integration of 2D material-based flexible AI-processing hardware that consists total of six layers of transistors and memristor arrays (Fig. 8f) [13]. The vertical M3D devices were realized by sequentially peeling and stacking AI processing layers fabricated with 2D materials on a flexible PI substrate coated on a rigid carrier wafer. The 2D material-based vertically staked M3D electronics consist of WSe_2_/h-BN-based memristor array and MoS_2_-based FET arrays which combined together to provide various functionalities including sensing, signal-processing, and computing. The developed 2D materials-based M3D integration technology with non-von Neumann-based near/in-sensor computing architecture provided efficient edge computing with data acquisition directly from the adjacent layers that enabled fast data processing and transmission with lower power consumption and lower latency. Furthermore, the low stiffness and low internal stress of 2D materials enabled the multi-stacking of various device tiers in a wafer-free manner on an ultrathin flexible polymeric support which provided exceptional mechanical flexibility and other merits to the developed device. The device exhibited excellent mechanical robustness under repetitive bending up to 100 cycles without noticeable performance degradation.

## Challenges and their mitigations

### High contact resistance

**Fig. 9:**
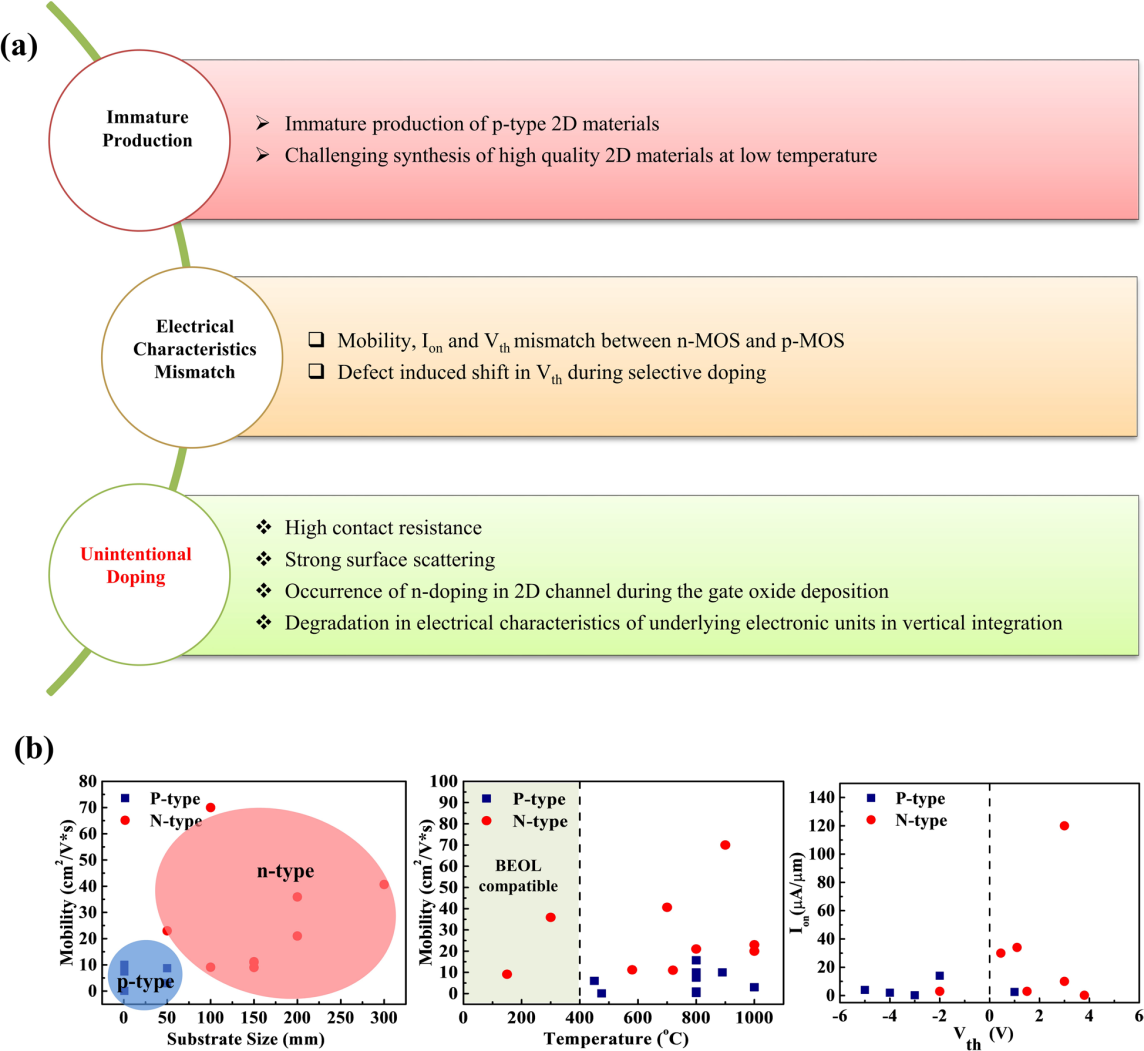
Challenges associated with the development of 2D materials-based CMOS. **a** Technological issues **b** Performance assessment of the p- and n-MOS units of the reported CMOS devices utilizing 2D materials

The ultralow thickness and dangling bonds-free surfaces make 2D materials a very promising alternative for developing high-performance CMOS electronics with greater integration density than conventional semiconductors can achieve. However, 2D materials possess several inherent limitations and challenges (Fig. 9a, b). For example, the existence of high contact resistance at the metal 2D semiconductor interface is a huge hurdle that demands dedicated efforts toward minimizing the contact resistance to realize effective CMOS devices [165–169]. In conventional bulk semiconductors, the issue of contact resistance can be very effectively managed by locally increasing the doping level, which lowers the barrier height at the metal–semiconductor interface by selectively modulating the Fermi level. However, in 2D material having ultra-thin thicknesses, the ion implantation-based doping is not viable, and the direct deposition of metal on the 2D materials surface also leads to the lattice damage that degrades the device performance due to the Fermi level pinning effect associated with the metal-induced gap states (MIGS) [165–168]. To tackle this challenge, several strategies have been investigated, which are generally successful up to some extent. For example, the insertion of a thin dielectric layer at the metal/2D interface is the most common approach, which induces surface charge transfer based n-doping at the contact area to enable the effective emission of carriers, additionally, the physical separation of the metal from the direct contact to the 2D materials surface effectively overcome the MIGS effect leading to effectively manage the contact resistance issue [59, 170]. Several other alternative approaches, such as instead of depositing metals, the transfer of metal patterns on 2D materials or vise-versa, exploiting metallic or semi-metallic phases of 2D materials as an alternative to metals as contact materials, the use of edge contacts with metals have also been investigated [120, 124, 168, 169, 171–175]*.*

### Challenging doping

As mentioned earlier in Sect. 6.1, the selective doping of a semiconductor at the contact area and at the channel region is an essential part of CMOS manufacturing, which relies on incorporating a controlled amount of the desired dopant in the intrinsic semiconductor. The selective doping at the contact region is essential to achieve low contact resistance, while doping at the channel region is necessary to ensure the required complementary electronic functionality. In contrast to the conventional bulk semiconductors, the traditional ion-implantation-based doping cannot be executed for 2D materials as their ultrathin crystal is vulnerable to damage from ion bombardment. Therefore, the alternative strategies that can provide effective modulation in charge carrier mobility without damaging the structure of 2D materials are essential to effectively exploit 2D semiconductors in CMOS electronics. The grafting of 2D material’s surface with self-assembled monolayers (SAMs) of specific organic compounds is one of the most facile and effective approach that can locally tune the doping type [176–180]. The treatment of 2D material surfaces with plasma and with particular chemicals such as dichloroethane, benzyl viologen (BV), and AuCl_3_, has also been explored [181–188]. While these techniques offer a straightforward method for tuning carrier polarity, their practical application in CMOS devices is constrained by the instability associated with chemical-based surface treatments. The passivation of 2D materials surface with an oxide-based dielectric material is a promising alternative to achieve a stable modulation in charge carrier polarity. Leveraging this approach, several studies have shown stable conversion between p- and n-type characteristics, particularly in TMDs, along with the successful fabrication of 2D semiconductor-based CMOS devices [59, 189–191].

Most of the surface-modification and passivation-based methods typically incorporate various contaminations and damage to the 2D material’s surface that consequently leads to the Coulomb scattering and thereby performance degradation. Consequently, developing strategies to adjust the doping type in 2D semiconductors with minimal or no damage to their surfaces would be a breakthrough. In this context, Lee et al. attempted to modulate the doping type in MoS_2_-based transistors fabricated in the form of WSe_2_/h-BN/MoS_2_ heterostructure [112]. The doping in the MoS_2_ channel was modulated remotely by controlling the charge transfer between WSe_2_ and MoS_2_, which was separated by an ultrathin 2D insulating barrier (h-BN). The charge density on the WS_2_ surface was adjusted by treating it with the different concentrations of n-type molecular dopant triphenylphosphine (PPh_3_). The designed 2D heterostructure-based TFTs resemble high electron mobility transistors (HEMTs), featuring spatial separation between charge carriers and surface dopants. This configuration allows for the development of a high-mobility transistor that confines charge transport in two dimensions while minimizing impurity scattering. The aforementioned study provides an excellent foundation for modulating doping without negatively impacting the device performance, which can be further extended to change the doping type to realize high-performance CMOS devices. In another study, Seo et al. reported the photo-induced reversible doping in few-layer MoTe_2_ and WSe_2_ and tuning the channel polarity from n- to p-type, and vice versa under the exposure of laser light of different wavelengths. Exploiting this approach, the CMOS device was fabricated on a single 2D material channel which exhibited the dynamic tuning in the circuit functionality via tuning the incident light frequency.

### Unwanted doping with dielectric deposition

The large area CMOS electronics consists of several MOSFETs as vital components which essentially demands the deposition of high-quality gate dielectric. The ALD technique is a widely used technique for the deposition of various high-k gate dielectric materials in modern silicon electronics. This method offers high-quality films with precise thickness control, excellent uniformity, and conformal coverage [192, 193]. However, the absence of dangling bonds on the surface of 2D materials complicates the use of the ALD process in 2D technology, as it lacks nucleation sites necessary for initiating chemical reactions [194–196]. To this end, various efforts have been made to successfully deposit the dialectic on the 2D material’s surface through surface pre-treatment to artificially create the defects as nucleation sites. For example, ozone plasma treatment on the MoS_2_ surface creates the lattice defect such as S vacancy or MoO bonds, that can act as activated dangling bonds for the ALD deposition on high k-oxides [197–199]. A thin buffer layer of certain polymers and molecules on top of the 2D materials surface has also been explored for accomplishing ALD growth [200, 201]. Although the above approaches provided a reasonable success, they experience several challenges. For example, the plasma treatment typically damages the 2D materials and thereby can degrade their electrical performance. The oxide-based gate dielectrics deposited on the 2D materials surface utilizing the organic buffer layer usually resulted in a lower dielectric constant that hampers the gate controllability. Furthermore, oxide-based dielectrics typically induce n-doping in 2D semiconductors through oxygen vacancy-mediated surface charge transfer. While this doping approach is appropriate to convert p-type 2D material into n-type for realizing n-channel TFTs of the CMOS devices, it is unsuitable for fabricating p-channel 2D TFTs. Alternatively, few vdW insulators e.g., CaF_2_, BN, and mica have been investigated as gate insulators that provide low trap states, minimal damage, and do not introduce undesirable doping to the underlying 2D materials [202–208]. The transferred vdW dielectric provides minimal damage to the underlying 2D materials, however, they suffer from poor scalability and inferior insulating properties leading to larger leakage currents which are undesired for low-power electronics.

**Fig. 10:**
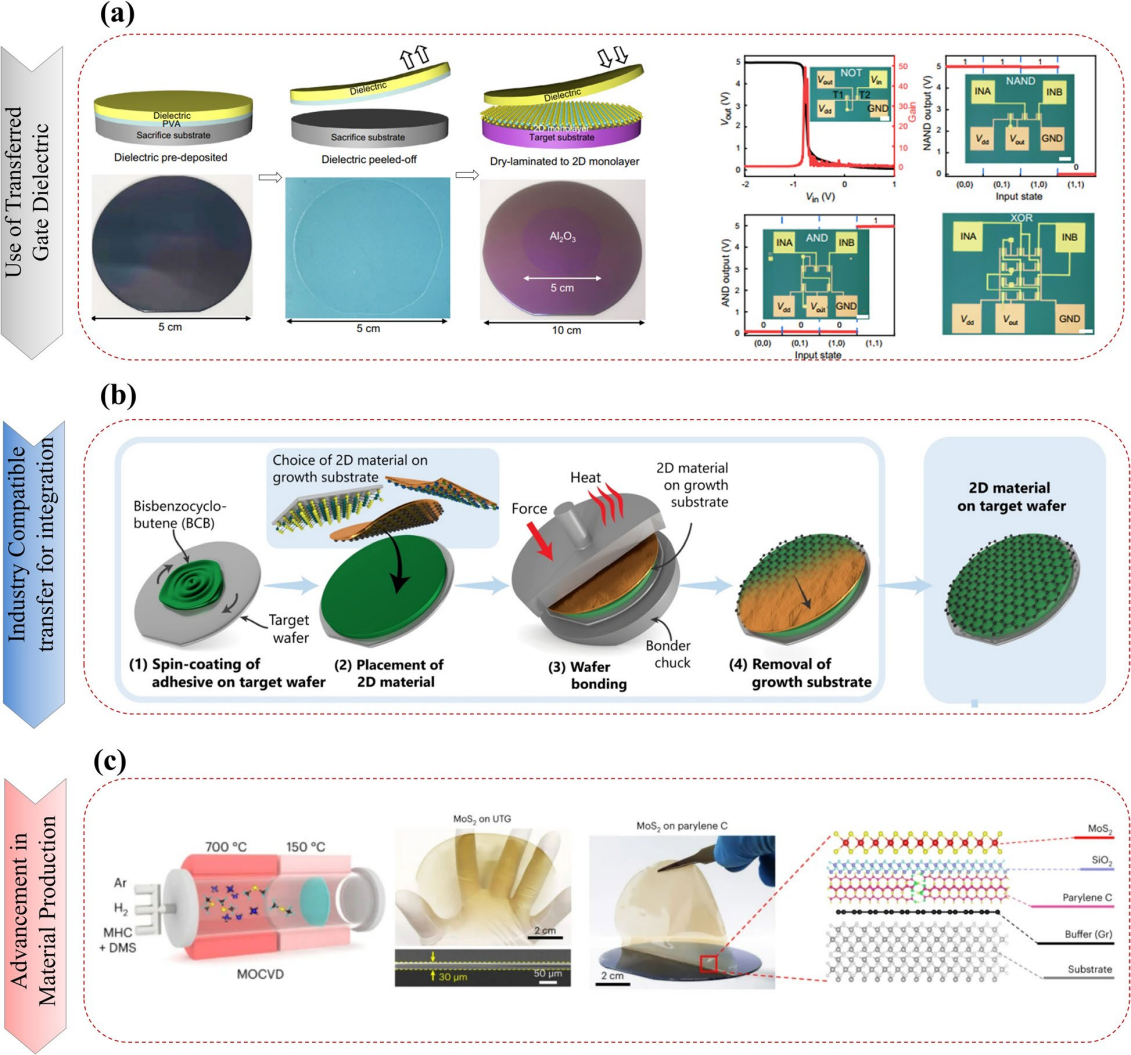
Strategies to address the challenges hindering the commercialization of 2D materials-based CMOS electronics. **a** The use of transferred gate dielectric for the realization of large-area devices with high performance. Schematics representation showing the process-flow for the transferring wafer scale gate dielectric. The below panel shows corresponding optical images. The right panel shows the optical image and corresponding output versus input-voltage characteristics of the fabricated logic devices. Reproduced with permission [210]. Copyright 2023, Springer Nature. **b** The industrial compatible 2D materials transfer process. Schematic representation of the process steps involved in the wafer scale transfer of 2D materials. Reproduced with permission [240]. Copyright 2021, Springer Nature. **c** The strategy to achieve a wafer-level synthesis of 2D material at low temperatures. Schematic representation of the synthesis chamber, the optical images of synthesized 2D materials (e.g., MoS_2_) on ultrathin glass and flexible polymeric substrates. Reproduced with permission [71]. Copyright 2023, Springer Nature

The integration of high-quality dielectrics on 2D materials surfaces over large scale remains a challenge. The recently developed strategy of depositing oxide-based dielectrics on a suitable rigid substrate followed by its transfer to use as a gate dielectric in the device fabrication provides a potential solution for large area, damage, and degradation-free intergradation of gate dielectric in 2D materials-based CMOS devices [209, 210]. For example, Lu et al. reported the integration of wafer scale high-k dielectric onto the 2D materials surface as gate dielectric using a dry transfer approach (Fig. 10a). They initially deposited Al_2_O_3_ or HfO_2_ dielectric films of desired thickness using the ALD technique onto a handling wafer coated with a 9 nm thick PVA as a sacrificial layer. The deposited dielectric films were then mechanically dry-released and dry-laminated on top of wafer-scale MoS_2_ monolayers, having a vdW gap at the interface, which helps in maintaining the intrinsic electrical properties of MoS_2_ and minimizes the carrier scattering in the channel. The ultrathin, large-area dielectric films integrated on top of the 2D material channel, exhibited the crack or damage-free morphology with wafer-scale flatness. The transferred dielectric film exhibited electrical characteristics similar to that of the as deposited one with the capacitance, equivalent oxide thickness (EOT), leakage current, and breakdown field of 2.8 μF/cm^2^, 1.2 nm, 10^–7^ A/cm^2^, and 6 MV/cm, respectively. The vdW interface between MoS_2_ and transferred Al_2_O_3_ dielectric enabled the fabrication of high-performance TFTs avoiding the charge-transfer-based doping effect in 2D channels. The fabricated TFTs exhibited excellent electrical characteristics with a high on–off ratio, low subthreshold swing, and low interface states of 10^7^, 68 mV/dec, and 7.6 × 10^9^ cm^−2^ eV^−1^, respectively. The fabrication of various logic circuits such as NOT, NAND, NOR, AND, and XOR gates was demonstrated by exploiting the high-performance TFTs designed with MoS_2_ and transferred Al_2_O_3_ dielectric.

### Challenges with transfer of 2D materials

At the early stage of the development, the 2D materials-based fundamental electronic units were demonstrated using the mechanical exfoliation and transfer of 2D materials on the target substrates. Later on, with the advancement in the CVD/MOCVD synthesis of large-area high-quality 2D semiconductors, the complex electronic circuits were realized by transferring the grown 2D materials on the desired substrates. Since most of the flexible electronic devices typically use low thermal budget substrates, directly growing high-quality 2D materials on such substrates using high-temperature CVD/MOCVD synthesis presents a challenge. The solution-based wet transfer of 2D materials from the growth substrate, using PMMA as a supporting film, is the most widely used method in fabricating the 2D materials-based electronic devices [211, 212]. The process involves spin-coating PMMA onto the grown 2D materials, followed by etching the growth substrate with an appropriate etchant, causing the PMMA/2D material stack to float on the surface of the etchant. After multiple intermediate soakings in deionized water, the floated 2D materials with PMMA support are transferred onto the desired substrates by scooping. Finally, the PMMA supporting film, which also protects the 2D materials from damage or degradation in the etchant solution, is removed using the appropriate solvent. The residue- and damage-free transfer of 2D materials onto arbitrary substrates with clean interface is crucial for high-performance electronic devices. However, in the PMMA-assisted wet transfer approach, the complete removal of PMMA is challenging [213]. To release the PMMA/2D material stake from the growth substrate, an acid-based substrate etching is commonly employed, which can potentially damage and degrade the quality of ultra-thin 2D materials. In this context, Shinde et al. developed a unique strategy of the complete water-based transfer of 2D materials [92]. In this approach, the SiO_2_ surface of the growth substrates were treated with diluted HF prior to the growth process, it allows the wafer-scale MoS_2_ thin films to float directly on the water surface without using acid-based etchants. This direct water-assisted transfer of MoS_2_ films allows the damage and degradation-free transfer onto the arbitrary substrates with fewer defects.

Although the solution-based wet transfer methods mentioned above offer a simple way to transfer large-area 2D materials onto arbitrary substrates, the complete removal of PMMA remains a challenge. Any residual PMMA left on the 2D material surface can lead to degradation of the electrical performance of the fabricated devices. Several polymer films such as 2-(diphenylphosphine) spiro-fluorene (SPPO1), polycarbonate (PC), pentacene, polyvinyl alcohol (PVA), rosin and paraffin have been attempted as an alternative to PMMA [214–219]. However, the complete removal of these films remains a challenge. In addition to the issue of polymer residues, the wet transfer process can also induce other mechanical damages such as the formation of wrinkles, bubbles, and edge curling in the transferred 2D materials. As an alternative to the wet transfer process, the dry transfer of 2D materials has also been explored. Polydimethylsiloxane (PDMS) and the thermal release tape (TRT) are the most frequently employed materials in the dry transfer process of 2D materials [220, 221]. In PDMS based dry transfer approach, releasing 2D materials from the PDMS surface onto the target substrates is challenging due to the difficulty in controlling the adhesion strength between the two interfaces. The dry transfer using TRT also faces the challenges of leaving adhesive residues on the surface of 2D materials after transfer. Although dry transfer with PDMS and TRT enables roll-to-roll-based, industrially compatible large-scale transfers, the mechanical damage to 2D materials during the process remains a significant challenge. To effectively utilize 2D materials in commercial applications, the automated fabrication aligned with the current Si foundry is necessary. In recent years, few attempts have been made to demonstrate the automated pick-up and transfer of 2D materials at desired locations with significant success [222–224]. However, the ultrathin nature of 2D materials, along with various mechanical and surface-related complexities, make automation in their transfer challenging. Further research in integrating advanced machine learning models and optimization processes is necessary for realizing the automated transfer of 2D materials at the commercial level.

### Challenges with flexible CMOS

Along with the material characteristics-induced intrinsic issues, the flexible CMOS devices developed with 2D materials face additional challenges. As most of the flexible substrate used in flexible electronics possesses high surface roughness and low thermal budget, which degrade the device performance and increase complexity in fabrication [225, 226]. In addition, these flexible polymeric substrates usually exhibit poor water vapor transmission ratio (WVTR) which adversely accelerates the oxidation in 2D materials and consequently leads to instability and performance degradation in the developed devices [227, 228]. The 2D materials-based flexible CMOS devices fabricated over large areas are usually designed in a multitasked manner including a substrate, the electronic components such as p- and n- MOSFETs, and a passivation layer. During the operation, these flexible devices usually experience various vicious deformations such as stretching, twisting, bending, and shearing in a repeated manner which potentially incorporate the strain and mechanical damage in the device units that subsequently leads to performance degradation with sliding, buckling, or delamination. The applied strain in the active semiconducting channel of the fabricated device can adversely affect the electrical properties of 2D materials, which can disrupt the device's functionality and integrity. Thus, to successfully integrate 2D materials into a practical flexible CMOS device, it is essential to address the intrinsic material-related issues together with the other challenges that occur due to the use of flexible polymeric substrates including limited processing temperatures, strain-induced failures, and subpar performance.

The performance degradation issue associated with high surface roughness and high WVTR can be substantially addressed by depositing a thin buffer layer of oxide materials such as SiO_2_ or Al_2_O_3_ on top of the flexible polymeric substrates [229–231]. To tackle the low thermal budget of the polymeric substrates including huge thermal expansion coefficient mismatch with 2D materials, the steps of device fabrication such as dielectric and metal deposition are typically performed under certain temperatures utilizing the ALD and PVD approaches [229, 232]. The use of transferred dielectric and metal electrodes instead of deposition is also a potential alternative. Instead of using a polymeric substrate, substrates such as mica and ultrathin glass (UTG) possessing higher temperature tolerance can also be exploited for applications where high flexibility or stretchability is not mandatory. The robust and reliable functionality of the flexible/bendable 2D materials-based CMOS device demands a device design that enables the incorporation of strains in a 2D channel under the fracture limit during operation. The use of thin substrates can offer a low bending radius while incorporating lower strain, however, using the flexible substrates beyond a certain thickness (typically 10–50 µm) is not feasible for the shake of effective handling. The innovative device designs in which active 2D materials and other strain-vulnerable layers e.g., gate insulators, encapsulations, and buffer layers are placed at the location of the neutral mechanical plane (NMP), represent a promising strategy offering decreased risk of strain-induced failure with increased mechanical robustness [233, 234].

### Challenges with 3D integration and commercialization

The 2D materials with dangling bonds-free surfaces have already demonstrated their potential to attain the ultimate limit of miniaturization in electronic devices by integrating them in a vertical direction. In recent years several investigations have been made demonstrating the integration of 2D materials on top of the Si CMOS and also the development of all 2D materials based M3D electronics with high integration density at laboratory scale [23, 24, 26, 162]. To capitalize the 2D materials at the industrial level, the integration of 2D materials with existing semiconductor manufacturing lines is essential. Advancements in CVD and MOCVD techniques have significantly improved the production of 2D materials up to the wafers as large as 8 inches with the material quality close to intrinsic levels [235]. However, the synthesis of good-quality 2D material usually requires a high processing temperature of more than 500 ºC, which makes it difficult to directly synthesize them on wafers containing pre-processed electronics such as CMOS or other BEOL components. Therefore, the vertical integration of 2D materials typically relies on material transfer from the growth substrates to the dedicated device substrate. The water-assisted wet transfer approach is the most popular method due to its facile process flow of placing the 2D material directly on the target locations [65, 92, 236–238]. Although the wet transfer method is simple, it can negatively affect the material’s properties by introducing defects, wrinkles, and strain in the transferred layer, along with residuals from the polymeric carrier layer remaining on the surface of the 2D materials. To transfer synthesized 2D materials from the growth substrates, alternative dry transfer techniques using stamps and laminators have also been attempted, however, these methods can also cause various damages including micro-cracks, wrinkles, and contamination associated with sacrificial carrier layers [239].

The industrial adaptation of 2D materials required the wafer-scale (100 to 300 mm wafers) transfer in alignment with standard semiconductor manufacturing processing while maintaining the prerequisite quality of 2D materials. In this context, Quellmalz et al. demonstrated an adhesive wafer bonding-based industrially compatible approach for the transfer and integration of wafer scale 2D materials onto the target substrates [240]. In this approach, the as-grown wafers of 2D materials were bonded with another wafer having a thermosetting polymer-based adhesive layer facing toward 2D materials (Fig. 10b). This sandwiched assembly of two wafers was then loaded in a wafer bonding tool for uniformly applying optimized pressure and temperature. Under these conditions, the viscosity of the adhesive layer decreases which enables the delamination of the 2D materials from the growth substrate in a highly controllable manner while incorporating minimal damage. Using this approach, a large area heterostructure stake of various 2D materials including graphene, MoS_2_, and h-BN was demonstrated. The fabricated encapsulated graphene field-effect device exhibited carrier mobility as high as 4520 cm^2^/Vs. The wafer bonding is a mature approach that is commonly employed in semiconductor manufacturing. Therefore, the demonstrated utilization of wafer bonding tool in the transfer of 2D materials can be an important step towards the development of 2D materials based vertically integrated high-density functional electronics. The low-temperature synthesis of high-quality 2D materials will be a very promising alternative for the development of high-performance vertically integrated M3D devices. If the specific 2D semiconductors such as MoS_2_ or WSe_2_ can be grown at low temperatures, particularly below 200 ºC, it would enable the realization of all 2D-based M3D integrated CMOS devices. In a recent study, Hoang et al. developed a strategy for the synthesis of MoS_2_ monolayers over 100 mm wafers at a temperature as low as 150 ºC using the MOCVD approach (Fig. 10c) [71]. The low-temperature synthesis enabled the fabrication of logic circuits and other devices directly on polymeric substrates, in which the material quality of MoS_2_ was maintained by avoiding the transfer process. The monolayer MoS_2_ synthesized directly at polymer substrates exhibited a field effect mobility of 9.1 cm^2^ V^−1^ s^−1^ and a positive threshold voltage of + 5 V, which is essential in realizing various applications with low power consumption. The demonstrated approach of low-temperature synthesis of 2D materials on various substrates laid a very promising platform for the realization of vertically integrated high-density M3D devices for various applications.

## Conclusion and outlook

Recent advances with 2D semiconductor-based FETs have paved the way for the development of high-performance miniaturized electronics, especially CMOS devices, with exceptional scalability and remarkable integration density. The extensive library of available p- and n-type 2D semiconductors, combined with strategies for locally modulating carrier polarity, make 2D materials a promising alternative to traditional semiconductors. The emerging era of artificial intelligence and the metaverse strongly demand the development of highly integrated devices such as graphic processing units (GPUs), high-bandwidth memory (HBM) and near-eye displays (NED), and others, that can handle large amounts of data [241, 242]. These devices typically require a high degree integration of low-dimensional electronic units having high performance. Silicon-based electronic integration has already reached its physical limits, facing critical challenges associated with the short channel effect and high processing temperatures, which hinder both the lateral and vertical integration beyond a certain dimensional scale. Due to their immunity to the short channel effect and their ability to retain superior electrical characteristics even at sub-nanometer thickness, the 2D materials are highly promising in developing electronics with high integration density [235, 243]. The International Roadmap for Devices and Systems (IRDS) has announced 2D materials as next-generation semiconductors, sparking significant interest, not only in academia but also in corporate research [202, 244–254]. The leading semiconductor companies such as Samsung**,** Intel, TSMC and IMEC have already started developing low-dimensional electronic units, such as transistors, with 2D semiconducting channels. For example, Samsung has reported the uniform growth of 2D semiconductors over 8-inch scale and demonstrated the successful fabrication of the transistor on grown 2D materials with a yield exceeding 99.97% [255]. Intel demonstrated a double-gated device with 10 nm channel length and low leakage current [251]. Whereas**,** IMEC demonstrated the fabrication of top gated transistors with a channel length below 5 nm [253]. Considering the importance of the 2D materials in the future electronics, several manufacturing companies such as Aixtron and Veeco are focusing on developing tools and equipment capable of producing 2D materials over large scale. Moreover, several material production companies, such as 2D Factory and Graphenea, have already demonstrated the industrial-scale production of 2D materials.

**Fig. 11:**
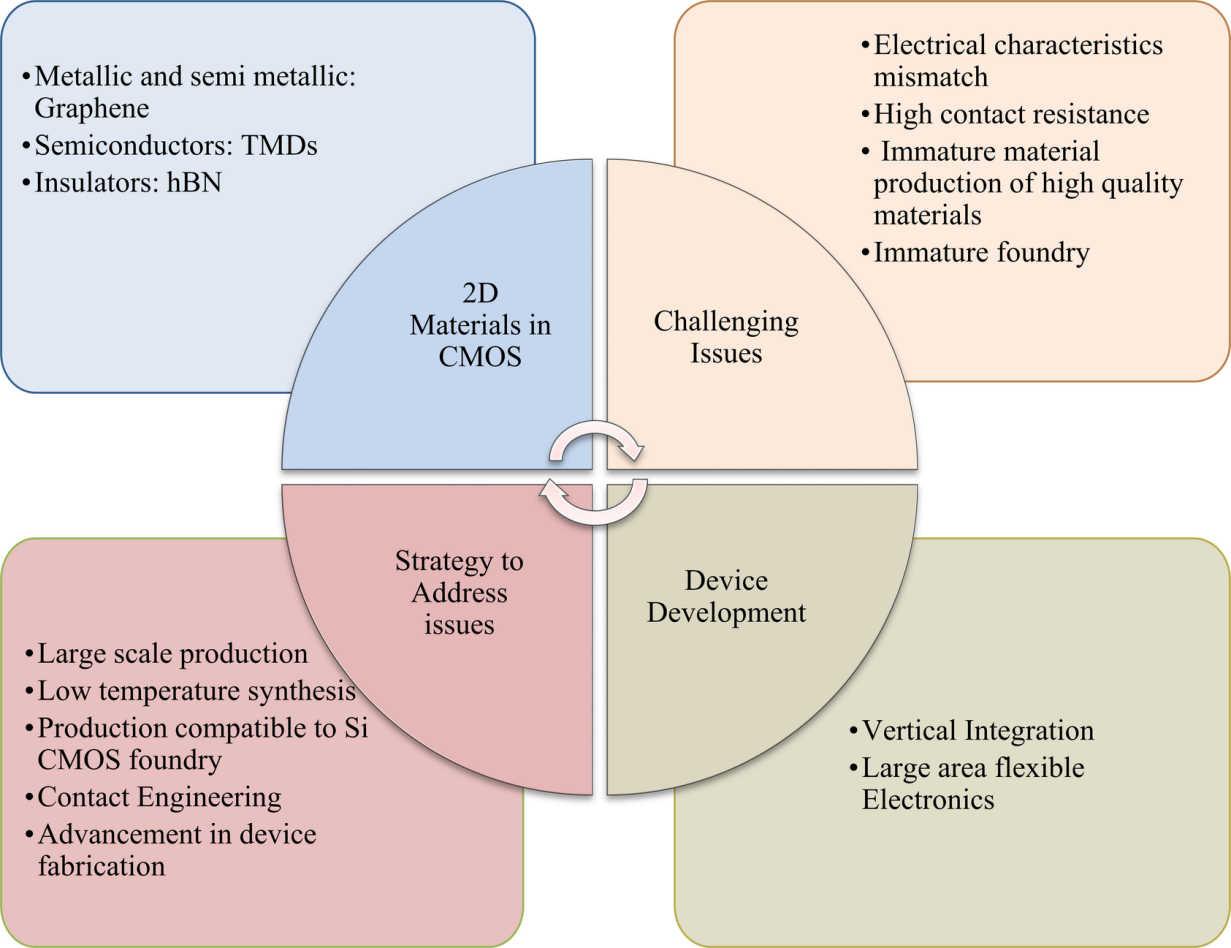
Different research paths for realizing 2D materials-based CMOS electronic at the industrial standard with highlighting associated challenges and potential strategies to overcome them

Although 2D materials have made significant advancements and demonstrated their potential in developing efficient CMOS devices, the commercial adaptation of 2D technology in multifunctional electronics is still in its early stages. The 2D materials typically possess few inherent limitations and challenges. For example, 2D materials-based electronic devices suffer from the high contact resistance issue, that can seriously impede the device's performance (Fig. 11). The high-quality gate dielectric, essential to realize a CMOS device, is challenging to deposit on the dangling bonds-free surface of 2D materials. Furthermore, the intricate process of selective doping for localized p- or n-type conversion poses additional challenges for CMOS electronics based on 2D materials. Moreover, low-temperature processing is necessary to integrate 2D materials with existing Si-CMOS electronics or to develop all 2D-based vertical M3D devices. However, the synthesis of a reasonable quality 2D material at a low thermal budget remains a challenge. Typically, the 2D materials-based M3D device relied on the synthesis of desired 2D materials at high temperature via the MOCVD approach followed by subsequent transfer to realize staked design. The transfer process is a crucial step in 2D materials-based device fabrication, and there is still much to be accomplished to achieve a defect- and damage-free transfer of 2D materials onto the desired substrate over large areas. The development of MOCVD synthesis for n-type materials, (particularly MoS_2_) on large-area wafers at low temperatures, has made significant progress. However, producing p-type 2D semiconductors still requires additional efforts to achieve the necessary yield with high-quality materials for commercial viability. In general, for 2D materials-based CMOS technology to achieve commercial success comparable to the Si CMOS industry, substantial efforts and innovative demonstrations in terms of device strategies and material production are still required.

## Data Availability

Not applicable.
